# Escape from TGF‐β‐induced senescence promotes aggressive hallmarks in epithelial hepatocellular carcinoma cells

**DOI:** 10.1002/1878-0261.70021

**Published:** 2025-03-14

**Authors:** Minenur Kalyoncu, Dilara Demirci, Sude Eris, Bengisu Dayanc, Ece Cakiroglu, Merve Basol, Merve Uysal, Gulcin Cakan‐Akdogan, Fang Liu, Mehmet Ozturk, Gökhan Karakülah, Serif Senturk

**Affiliations:** ^1^ Izmir Biomedicine and Genome Center Turkey; ^2^ Department of Genomics and Molecular Biotechnology, Izmir International Biomedicine and Genome Institute Dokuz Eylul University Izmir Turkey; ^3^ Department of Biomedicine and Health Technologies, Izmir International Biomedicine and Genome Institute Dokuz Eylul University Izmir Turkey; ^4^ Center for Advanced Biotechnology and Medicine, Susan Lehman Cullman Laboratory for Cancer Research, Ernest Mario School of Pharmacy, Rutgers Cancer Institute of New Jersey Rutgers, The State University of New Jersey Piscataway NJ USA; ^5^ Department of Medical Biology Izmir Tinaztepe University School of Medicine Turkey; ^6^ Department of Molecular Biology and Genetics Bilkent University Ankara Turkey

**Keywords:** EMT, hepatocellular carcinoma, resistance, senescence escape, TGF‐β

## Abstract

Transforming growth factor‐β (TGF‐β) signaling and cellular senescence are key hallmarks of hepatocellular carcinoma (HCC) pathogenesis. Despite provoking senescence‐associated growth arrest in epithelial HCC cells, elevated TGF‐β activity paradoxically correlates with increased aggressiveness and poor prognosis in advanced tumors. Whether the transition between these dichotomous functions involves modulation of the senescence phenotype during disease progression remains elusive. Exploiting the epithelial HCC cell line Huh7 as a robust model, we demonstrate that chronic exposure to TGF‐β prompts escape from Smad3‐mediated senescence, leading to the development of TGF‐β resistance. This altered state is characterized by an optimal proliferation rate and the acquisition of molecular and functional traits of less‐differentiated mesenchymal cells, coinciding with differential growth capacity in 2D and 3D culture conditions, epithelial‐to‐mesenchymal transition (EMT), and increased invasiveness *in vitro*, and metastasis *in vivo*. Mechanistically, resistant cells exhibit defective activation and nuclear trafficking of Smad molecules, particularly Smad3, as ectopic activation of the TGF‐β/Smad3 axis is able to reinstate TGF‐β sensitivity. An integrated transcriptomic landscape reveals both shared and distinct gene signatures associated with senescent and TGF‐β resistant states. Importantly, genetic ablation and molecular studies identify microtubule affinity regulating kinase 1 (MARK1) and glutamate metabotropic receptor 8 (GRM8) as critical modulators of the resistance phenomenon, potentially by impairing spatiotemporal signaling dynamics of Smad activity. Our findings unveil a novel phenomenon wherein epithelial HCC cells may exploit senescence plasticity as a mechanism to oppose TGF‐β anti‐tumor responses and progress towards more aggressive HCC phenotypes.

## Graphical abstract

Chronic TGF‐β exposure drives epithelial HCC cells from a senescent state to a TGF‐β resistant mesenchymal phenotype. This transition is characterized by the loss of Smad3‐mediated signaling, escape from senescence, enhanced invasiveness and metastatic potential, and upregulation of key resistance modulators such as MARK1 and GRM8, ultimately promoting HCC progression.

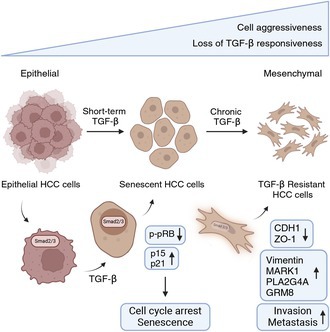

**Abbreviations**
AMPK: AMP‐activated protein kinase
BrdU: bromodeoxyuridine
C12FDG: 5‐dodecanoylaminofluorescein di‐beta‐d‐galactopyranoside
cAMP: cyclic AMP
CDH1: E‐cadherin
Doxo: doxorubicin
dpf: days post‐fertilization
ECM: extracellular matrix
EMT: epithelial‐to‐mesenchymal transition
FDR: false discovery rate
FUCCI: fluorescence ubiquitination‐based cell cycle indicator
GRM8: glutamate metabotropic receptor 8
GSEA: gene set enrichment analysis
HCC: hepatocellular carcinoma
Huh7‐S: TGF‐β‐induced senescence
Huh7‐TR: TGF‐β resistant
MARK1: microtubule affinity regulating kinase 1
NES: normalized enrichment scores
PCA: principal component analysis
PKA: protein kinase A
PLA2G4A: phospholipase A2 group IVA
SARA: Smad anchor for receptor activation
SASP: senescence‐associated secretory phenotype
SA‐β‐Gal: senescence‐associated β‐galactosidase
SD: standard deviation
TGF‐β: transforming growth factor‐β
TPM: RSEM‐derived transcript per million
VIM: vimentin
ZO‐1: zonula occludens‐1

## 1 Introduction

Cellular senescence, the state of cell cycle arrest triggered by both intrinsic and extrinsic stressors, is a vital defense mechanism against further expansion of premalignant cells. Historically perceived as a static anti‐tumor phenomenon, recent evidence highlights the dynamic and plastic nature of senescence caused by oncogenic stress or cancer therapies, which can paradoxically promote cancer reprogression under specific circumstances [1, 2]. The transition from a tumor‐suppressive state to a tumor‐promoting phenotype can manifest through diverse processes, including the secretion of pro‐tumorigenic senescence‐associated secretory phenotype (SASP) factors from senescent cells, evasion of the senescence program, or reentry into the cell cycle by previously senescent cells [3, 4, 5]. Thus, the plasticity of tumor cells to modulate this cytostatic barrier presents significant implications for cancer progression and therapy resistance.

Transforming growth factor‐β (TGF‐β) is a multifunctional cytokine that governs a number of physiological and pathophysiological processes and functions as a potent inducer of cellular senescence across various biological contexts. Compelling evidence indicates that TGF‐β‐induced senescence occurs as a natural tumor‐suppressive response in multiple cancers, recapitulated with unique examples in lymphoma, lung cancer, and hepatocellular carcinoma (HCC) [6, 7, 8]. Aberrant TGF‐β signaling and cellular senescence have emerged as central modulators of sustained hepatic inflammation, fibrosis, and cirrhosis, collectively driving the initiation and progression of HCC [9, 10, 11]. The seminal study conducted in epithelial HCC cells, which mimic early forms of HCC tumors, illustrates that TGF‐β can provoke robust senescence and anti‐tumor responses *in vitro* and *in vivo* [12]. This emphasizes the potential of targeting the TGF‐β/senescence axis as a therapeutic approach, at least in a subset of patients with HCC. However, the apparent paucity of senescence in advanced tumors implies that tumor cells evolve mechanisms to oppose this phenomenon. Indeed, in advanced tumors, TGF‐β signaling appears to promote aggressive cancer cell traits, such as epithelial‐to‐mesenchymal transition (EMT), reshaping the tumor microenvironment and enhancing angiogenesis and metastasis [13, 14]. This is consistent with the fact that, despite its role as a tumor suppressor in premalignant hepatocytes and during the early stages of the tumor, chronic exposure to TGF‐β signaling is linked to tumor cell plasticity, stemness, and the progression and unfavorable prognosis of HCC [15, 16]. This transition is particularly evident *in vivo* and convincingly phenocopied *in vitro* in a large cohort of mesenchymal HCC cell lines that maintain a partially intact TGF‐β signaling yet demonstrate near complete resistance to TGF‐β‐induced cytostasis [17].

The dichotomous role of TGF‐β signaling in cancer is well documented; however, the mechanistic aspects governing this transition and whether the TGF‐β/senescence axis plays a fundamental role in this context remain poorly defined. A plausible hypothesis for this phenomenon postulates that, as the disease progresses, tumor cells adapt to mitigate the growth‐suppressive effects of TGF‐β‐induced senescence and acquire TGF‐β insensitive cellular states. Here, we interrogate this hypothesis in the epithelial HCC cell line model, Huh7. Initially, we characterize that senescence induction by TGF‐β is mediated through canonical Smad3 signaling. We next show that chronic TGF‐β exposure promotes subpopulations of Huh7 cells to overcome this growth‐suppressive senescent state, leading to the emergence of a persistent clone with full proliferative capacity. Notably, this subclone, dubbed Huh7‐TR (TGF‐β resistant), not only becomes resilient to TGF‐β‐mediated cytostasis but also acquires molecular and functional hallmarks of mesenchymal cells both *in vitro* and *in vivo*. At the molecular level, the resistance phenotype is associated with impaired TGF‐β/Smad signaling dynamics. Comparative transcriptome profiling unveils distinct gene signatures associated with cellular senescence and the development of insensitivity to TGF‐β. Molecular studies identify MARK1 and GRM8 as critical mediators of TGF‐β/senescence resistance, potentially by modulating activation and nucleocytoplasmic trafficking of Smad3. Furthermore, a similar transition is observed in the Hep3B cell line with comparable resistance and phenotypic changes, suggesting that this phenomenon is not unique to Huh7 cells. Thus, HCC tumor cells may exploit senescence as an adaptive mechanism to neutralize TGF‐β anti‐tumor responses and evolve into aggressive disease states.

## 2 Methods

### 2.1 Cell culture and treatments

The well‐differentiated HCC cell lines Huh7 (RRID:CVCL_0336) and Hep3B (RRID:CVCL_0326) were kindly provided by M. Ozturk (Izmir Tinaztepe University School of Medicine, Izmir, Türkiye). The HEK293T (RRID:CVCL_0063) cell line was kindly gifted by N. Ozturk (Gebze Technical University, Kocaeli, Türkiye). All cell lines have been authenticated by performing STR analysis and matching to reference STR profiles at 24 different loci plus the gender‐determining locus. Cell lines were grown in high glucose DMEM (Dulbecco's Modified Eagle's Medium, 41965039; Gibco, Paisley, Scotland, UK) with the addition of 10% fetal bovine serum (FBS; 10500064; Gibco, Paisley, Scotland, UK) and 1% penicillin/streptomycin (15140122; Gibco, Grand Island, NY, USA) at 37 °C and 5% CO_2_ culture conditions. Cells were routinely tested for mycoplasma contamination with the Mycoplasma PCR Detection Kit (G238; ABM, Richmond, Canada) and all experiments were performed with mycoplasma‐free cells. HEK293‐derived recombinant human TGF‐β1 ligand (100‐21; Peprotech, Cranbury, NJ, USA), hereafter referred to as TGF‐β, was reconstituted in 10 mm citric acid (pH 3.0), aliquoted, and stored at −80 °C until used. TGF‐β treatments were conducted with a ligand concentration of 5 ng·mL^−1^ unless specified otherwise in the figures. Huh7‐TR cells were derived by continuously exposing Huh7 cells to 5 ng·mL^−1^ TGF‐β for more than 50 days. Similarly, the Hep3B‐TR clone was derived from the Hep3B cell line following the same protocol. Cryo‐stock vials were made, and low‐passage cultures were used for all experiments. Cell treatments were performed with indicated doses of TGF‐β in complete media (unless otherwise stated) for indicated time points.

### 2.2 Transfection, lentivirus production, and cell transduction

pcDNA3 Flag‐Smad3‐WT was kindly gifted by K. Matsuzaki [18]. Huh7 and Huh7‐TR cells were transiently transfected with wild‐type Smad3 using Fugene‐6 Transfection Reagent (Roche, IN, USA). Transfections were conducted in 6‐well plates with an optimized Fugene‐6‐to‐DNA ratio (3 : 1). Briefly, cells were seeded at a density of 2 × 10^5^ per well to reach the desired confluency on the day of transfection. Transfected cells were incubated for 12–48 h until the time of the assay. HEK293T cells were used for lentivirus production. Cells were seeded in a 6‐well plate to reach approximately 80% confluency within 24 h. Co‐transfection was conducted with a target vector and the helper plasmids, psPAX2 (Addgene plasmid #12260; Addgene, Watertown, MA, USA) and pMD2.G (Addgene plasmid #12259; Addgene, Watertown, MA, USA), using a 4 : 2 : 1 ratio and a total DNA amount of 6 μg. PEI (Polyscience, 23966, Warrington, PA, USA) was utilized as the transfection reagent at a 1 : 5 DNA‐to‐PEI ratio. Two days after transfection, supernatants were filtered through a 0.45 μm SFCA filter (431220; Corning, NY, USA). The complete DMEM mixture containing viral particles and 8 μg·mL^−1^ Polybrene (H9268; Sigma, St. Louis, MO, USA) was added to target cells that were seeded at 50–60% confluency. Infected cells were selected with 2.5 μg·mL^−1^ puromycin (ant‐pr‐1; Invivogen, San Diego, CA, USA) for 3 days or by using fluorescence‐associated cell sorting (FACS) at FACSAriaIII (BD Biosciences, CA, USA).

### 2.3 RNAi silencing

RNAi silencing of target genes was performed following a previously published protocol [19]. pRetroSuper‐GFP‐Smad2 (Addgene plasmid #15722; Addgene, Watertown, MA, USA), pRetroSuper‐GFP‐Smad3 (Addgene plasmid #15723; Addgene, Watertown, MA, USA), and pRetroSuper‐GFP‐Smad4 (Addgene plasmid #15724; Addgene, Watertown, MA, USA) retroviral vectors were used to silence Smad protein expression. The Luc‐pSUPER‐Retro‐GFP/Neo vector (Addgene plasmid #22133; Addgene, Watertown, MA, USA) was used as a non‐targeting control. pLP/VSVG (K497500; Thermo Scientific, Carlsbad, CA, USA) and pCL‐Eco (Addgene plasmid #12371; Addgene, Watertown, MA, USA) helper plasmids were employed for retrovirus packaging. Target vector:pLP/VSVG:pCL‐Eco was blended in an optimal 1 : 1 : 1 weight‐to‐weight ratio. Following retroviral transduction, infected Huh7 cells were enriched for GFP‐positive cells via FACSAriaIII (BD Biosciences, CA, USA). The efficiency of knockdown was confirmed by western blotting and qRT‐PCR. Lentiviral shRNA construct targeting human GRM8 (TRCN0000219406) was established as previously described [20]. Briefly, hairpin oligonucleotides with AgeI (R3552S; NEB, Ipswich, MA, USA) and EcoRI (R3101S; NEB, Ipswich, MA, USA) restriction site overhangs were designed and synthesized. The oligonucleotides used are provided in Table S1. Annealed oligos were ligated into the pLKO.1 puro backbone (Addgene plasmid #8453; Addgene, Watertown, MA, USA), predigested by AgeI and EcoRI. Scrambled shRNA vector (Addgene plasmid #1864; Addgene, Watertown, MA, USA) was used as a non‐targeting control. E‐cadherin knockdown was performed using a publicly available vector (Addgene plasmid #18801; Addgene, Watertown, MA, USA).

### 2.4 Construction of lentiviral overexpression vectors

Wild‐type Smad3 coding sequence was inserted into the pLV‐EF1α‐IRES‐Puro vector (Addgene plasmid #85132; Addgene, Watertown, MA, USA). The Venus gene was excised from the pEF1α‐Cas9‐P2A‐Venus (pECPV) backbone [21, 22] and subcloned into pLV‐EF1α‐IRES‐Puro. For ectopic expression of TGFβRI, the rat TGFβRI coding sequence was extracted from the pRK5 TGF beta type I receptor Flag backbone (Addgene plasmid #14831; Addgene, Watertown, MA, USA) using EcoRI (R3101S; NEB, Ipswich, MA, USA) and HindIII (R3104S; NEB, Ipswich, MA, USA), and inserted into pEGFP‐C2 (6083‐1; Clontech, San Jose, CA, USA). The pEGFP‐C2 TGFβR1 vector was then digested with EcoRI (R3101S; NEB, Ipswich, MA, USA) and BglII (R0144S; NEB, Ipswich, MA, USA), and the insert was ligated into the pHIV‐Zsgreen vector (Addgene plasmid #18121; Addgene, Watertown, MA, USA), predigested with EcoRI and BamHI (R3136S; NEB, Ipswich, MA, USA). Huh7 and Huh7‐TR cells were individually infected with either the empty vector or the Smad3 or TGFβRI overexpression vector. Zsgreen‐positive cells were enriched via FACSAriaIII (BD Biosciences, CA, USA). Validation of Smad3 or TGFβRI overexpression was conducted through western blotting. FastFUCCI‐expressing polyclonal subline was established using pBOB‐EF1‐FastFUCCI‐Puro (Addgene Plasmid #86849; Addgene, Watertown, MA, USA) and packaging plasmids as described in transfection, lentivirus production, and cell transduction.

### 2.5 gRNA cloning

Oligonucleotides, synthesized by Macrogen (Seoul, South Korea), are provided in Table S1. Renilla luciferase gene‐specific gRNA (Ren g208) was used as a non‐targeting control. gRNA cloning was performed as described [22]. Briefly, oligonucleotide pairs for each gRNA were annealed, phosphorylated, and ligated into the pECPV plasmid, digested and dephosphorylated with FastDigest BsmBI (Esp3I; FD0454; Thermo Scientific, Vilnius, Lithuania) and FastAP Thermosensitive Alkaline Phosphatase (EF0651; Thermo Scientific, Vilnius, Lithuania), respectively. Ligation products were transformed into the Stbl3 strain (Invitrogen, CA, USA). Single colonies grown overnight on LB agar plates with ampicillin selection were validated through colony PCR. Successful plasmids for gRNA insertion were further purified (740588; Macherey Nagel, Düren, Germany) and sequence‐verified by Sanger sequencing. The efficiency of GRM8 knockout (KO) was assessed using the T7 Endonuclease I (T7E1) assay. Genomic DNA (gDNA) was extracted with the Quick‐DNA Miniprep Plus Kit (D4068; Zymo Research, Tustin, CA, USA), and the targeted GRM8 gene region was amplified by PCR using the following primers: GRM8‐F (5′‐TGATGGAAATTCACTGTGACATTCA‐3′) and GRM8‐R (5′‐ACACCAAAAGGGTCTATGAGCACCT‐3′). PCR products were separated by agarose gel electrophoresis and purified using the NucleoSpin Gel and PCR Clean‐up Kit (740609; Macherey Nagel, Düren, Germany). The assay was conducted according to the manufacturer's instructions (M0302; NEB, Ipswich, MA, USA).

### 2.6 RNA extraction and qRT‐PCR

Total RNA was isolated using the NucleoSpin RNA kit (740955, Macherey Nagel, Düren, Germany). RNA extracts were stored at −80°C. One microgram of total RNA was converted to single‐strand cDNA using the iScript cDNA Synthesis Kit (1708891; Bio‐Rad, Hercules, CA, USA). Corresponding cDNAs were subjected to quantitative reverse transcription PCR (qRT‐PCR) in triplicate, using gene‐specific primer pairs and BlasTaq 2× qPCR MasterMix (G892; ABM, Richmond, Canada). Relative expression of target mRNAs was normalized by housekeeping genes (GAPDH and β‐Actin) using the $2^{\Delta \Delta C_{t}}$ method. The primer pairs are provided in Table S2.

### 2.7 Subcellular fractionation

A total of 0.5–1 × 10^6^ cells were seeded in 10 cm plates, grown for 24 h, and then treated with 5 ng·mL^−1^ TGF‐β or complete DMEM for 60 min. Cells were rinsed three times with ice‐cold 1× PBS and scraped with 1 mL PBS into a 1.5 mL centrifuge tube. Cells were pelleted by centrifugation at 1500 ***g*** for 3 min. The pellet was resuspended in 300 μL Buffer A (10 mm HEPES pH 7.9, 10 mm KCl, 0.1 mm EDTA, 0.1 mm EGTA, 1 mm DTT, 0.5 mm PMSF) and mixed gently with a pipet tip. Cells were kept on ice for 15 min, then 25 μL 10% NP‐40 was added, vortexed vigorously for 10 s, and centrifuged at 11,000 ***g*** for 30 s. Supernatant (cytoplasmic fraction) was transferred into a new centrifuge tube and stored at −80°C. For the nuclear fraction, the pellet was resuspended in 50 μL ice‐cold Buffer C (20 mm HEPES, pH 7.9, 0.4 m NaCl, 1 mm EDTA, 1 mm EGTA, 1 mm DTT, with freshly added 1 mm PMSF). Samples were vigorously rocked for 15 min at 4°C and centrifuged at 11,000 ***g*** for 5 min. Supernatant (nuclear fraction) was transferred into a new centrifuge tube and stored at −80°C.

### 2.8 Western blotting

Western blotting was performed following a previously published protocol [20]. Briefly, cells were rinsed with ice‐cold PBS, scraped with modified RIPA Buffer (50 mm Tris pH 7.4, 150 mm NaCl, 1% Triton X‐100, 0.5% Na‐deoxycholate, 0.1% SDS, 1 mm EDTA, 1 mm EGTA, 10 mm β‐glycerophosphate, 10 mm NaF, 1 mm PMSF, 1 mm Na‐orthovanadate) containing Leupeptin, Aprotinin, and Pepstatin (10 μg·mL^−1^ concentration each), and lysed for 30 min on ice. Total protein concentrations were quantified using the Pierce BCA Protein Assay Kit (23227; Thermo Fisher Scientific, Rockford, IL, USA). Twenty microgram protein was separated on 10% SDS/PAGE gels and wet‐transferred onto 0.20 μm nitrocellulose membranes (10600004; GE Healthcare, Germany) at 350 mA for 90 min. Membranes were blocked with 5% (w/v) skim milk at room temperature for 1 h. Primary and secondary antibodies were diluted in 1% BSA in TBS‐T. Primary antibody incubations were carried out overnight at 4°C. Secondary antibodies were incubated for 1 h at room temperature. Details for primary and secondary antibodies are provided in Table S3. Rabbit antiserums against phospho‐Smad3 linker region residues (T179, S204 and S208) were kindly gifted by F. Liu (Rutgers, The State University of New Jersey) [23]. Images were obtained using the Odyssey Clx imaging system and image studio software (LI‐COR, Lincoln, NE, USA). β‐Actin or α‐tubulin was used as loading controls. BLUelf Prestained Protein Ladder (PM008/0500; GeneDirex, New Taipei City, Taiwan) was used as a protein marker.

### 2.9 Immunofluorescence staining

Cells were fixed with 4% formaldehyde for 10 min and permeabilized with 0.5% Triton X‐100 for 5 min. Blocking was done with 5% BSA in 1× PBS for 1 h at room temperature, followed by incubation with Smad‐specific primary and Alexa Fluor 488 or Alexa Fluor 568 secondary antibodies for 1 h at room temperature. Nuclear counterstaining was achieved with DAPI (1322MG005; Neofroxx, Einhausen, Germany). High‐resolution images were obtained using confocal microscopy (LSM880; Zeiss, Germany) or apotome fluorescence microscopy (Observer 7; Zeiss, Germany). Three independent researchers analyzed signals from multiple fluorescence images (at least 3 for each condition) in a double‐blind manner. Signal intensities were computed using a semi‐quantitative scoring metric, employing a simple method. Nuclear staining was scored on a 4‐point scale (0–3): scores of 1 and 2 indicated weak and moderate nuclear staining, respectively, both with some degree of cytoplasmic staining, while a score of 3 indicated strong nuclear staining with no apparent cytoplasmic signal. A score of 0 represented no detectable nuclear staining. C12FDG staining was performed as previously described [24]. Briefly, cells were collected and incubated in suspension with 33 μm C12FDG (5‐dodecanoylaminofluorescein di‐beta‐d‐galactopyranoside; HY‐126839; MedChemExpress, NJ, USA) diluted in regular culture media for 1 h in a cell culture incubator.

### 2.10 Cell proliferation assays

Cells were plated on glass slides in 12‐well plates at a density of 12 000 cells per well and treated with 5 ng·mL^−1^ TGF‐β or complete DMEM for 72 h. At 48 h into TGF‐β treatment, cells were incubated with 30 μm BrdU (B5002; Sigma Aldrich, St. Louis, MO, USA) for 24 h. Labeled cells were fixed in ice‐cold 70% ethanol for 10 min on ice. After DNA denaturation with 2 N HCl for 30 min, slides were rinsed with PBS‐T (0.1% Tween 20) for 5 min. Slides were incubated with BrdU monoclonal antibody (1/1000; 5292; Cell Signaling Technology, Danvers, MA, USA) for 2 h at room temperature, followed by labeling with either donkey anti‐mouse Alexa Fluor 488 (ab150105; Abcam, Cambridge, UK) or goat anti‐mouse Alexa Fluor 594 (8890S; Cell Signaling Technology, Danvers, MA, USA) for 1 h at room temperature. Cells were counterstained using DAPI for 1 min. Coverslips were rinsed and mounted onto glass slides. Images were acquired with a fluorescence microscope (BX61; Olympus, USA). The ratio of proliferating cells was assessed by dividing BrdU‐positive cells by the total number of DAPI‐stained cells. Senescence‐associated β‐galactosidase (SA‐β‐Gal) assay was performed as previously described with slight modifications [17]. Cells were seeded into 12‐well plates at a density of 12 000 cells per well and allowed to grow for 72 h (or seeded 8000 cells per well and allowed to grow for 120 h) in the absence or presence of TGF‐β. Cells were fixed with fixative solution (0.2% glutaraldehyde solution, 1.85% formaldehyde in PBS) for 10 min at room temperature. After rinsing with PBS, cells were incubated with staining solution (40 mm citric acid/Na phosphate buffer, 5 mm potassium ferrocyanide, 5 mm potassium ferricyanide, 150 mm NaCl, 2 mm MgCl_2_, 1 mg·mL^−1^ X‐gal) for 6 h at 37°C. When the blue color was fully developed, staining was monitored under a phase‐contrast microscope (IX71; Olympus, USA). For cell cycle analysis, cells were collected by trypsinization and fixed with ice‐cold 70% ethanol for 15 min on ice. Fixed cells were resuspended in 70 μL PI‐solution (50 μg·mL^−1^ propidium iodide, 0.1 mg·mL^−1^ RNase A and 0.05% TritonX‐100), and incubated for 40 min at 37°C by vortexing every 10 min. Following centrifugation at 3,500 ***g*** for 5 min at 4°C, stained cells were transferred to polystyrene tubes in 100 μL PBS and analyzed using a flow cytometer (LSRFortessa; BD Biosciences, CA, USA). Cell cycle distribution was assessed using flowjo v10.8.0 software (BD Biosciences, CA, USA).

### 2.11 Clonogenicity assays

For two‐dimensional (2D) colony formation assay, cells were seeded into 6‐well plates at 1000–1500 cells per well and treated with 5 ng·mL^−1^ TGF‐β or complete DMEM for 14–25 days, during which the medium was replaced every 3 days. Colonies were fixed with 3.7% formaldehyde (vol/vol) for 20 min and labeled with crystal violet solution (0.5% wt/vol crystal violet powder dissolved in 20% ethanol) for 40 min at room temperature. After full decolorization by thorough rinsing with tap water, plates were air‐dried at room temperature. High‐resolution images of colonies were captured using the Odyssey CLx Imaging System, and crystal violet signal intensities were quantified using imagej (National Institutes of Health, Bethesda, MD, USA) software. The three‐dimensional (3D) anchorage‐independent soft agar growth assay was performed as described [20]. Briefly, the bottom of the 12‐well plate was coated with a 0.5% noble agar (wt/vol) (J10907‐100gr; Alfa Aesar, Kandel, Germany) layer and left to solidify at room temperature for 30 min. Eight thousand cells in complete DMEM were mixed with agar (final concentration, 0.35%) and added to the top of the 0.5% agar layer. After the agar was solidified, DMEM supplemented with or without TGF‐β was added to each well, and the plate was placed in the incubator for 3 weeks. The medium was refreshed every 3 days. Images were obtained under a light microscope (CKX41; Olympus, USA) at 40× magnification. Five random fields per well were quantified according to the median diameter threshold of control cells. One representative colony from each condition was imaged under a phase‐contrast microscope (IX71; Olympus, USA) at 100× and 200× magnification. The hanging drop cell culture method (spheroid formation assay) was performed following a previously published protocol [20]. Briefly, cells were diluted in complete cell culture media supplemented with or without TGF‐β to achieve a concentration of 1000 cells/20 μL per droplet. Droplets were pipetted on the lid of a 10 cm tissue culture dish. The dish was filled with 1× PBS to avoid the evaporation of droplets. The lid was inverted on the dish, and the spheroids were incubated for 3 days. Images were obtained with a Stereo microscope equipped with 6.3× zoom. The average size of spheroid areas between different experimental conditions or cell clones was calculated using imagej software.

### 2.12 Wound healing and transwell invasion assays

Experiments were performed in duplicate with two wounds in each well. Cells were seeded in 12‐well plates, and a linear wound was made using a p200 pipette tip in confluent monolayer cells. Time 0 (zero) images were captured with a phase‐contrast microscope at 20× magnification. Cells were then cultured with or without TGF‐β in a growth medium containing 2% FBS. Scratches were photographed periodically. At least three measurements were used to calculate motility behavior. For the transwell invasion assay, inserts (353097; Falcon, NC, USA) were coated with a mixture of 0.25 mg·mL^−1^ Matrigel matrix (356234; Corning, MA, USA) and serum‐free DMEM (1 to 12.5 ratio, vol/vol) in a final volume of 120 μL for each well and incubated for 2 h at 37°C. A total of 6 × 10^4^ cells in 500 μL serum‐free DMEM were loaded onto the upper well of the transwell chamber. The lower well was filled with 750 μL of DMEM containing 10% FBS, and the cells were incubated for 24 h. Cells were fixed with 3.7% formaldehyde for 2 min. After permeabilization with 100% methanol for 20 min, chambers were stained with crystal violet for 15 min. Non‐invasive cells were carefully scraped off with cotton swabs. Invasive cells were imaged under a light microscope at 10× magnification and manually counted.

### 2.13 SEM imaging

Huh7 cells were seeded on 18 mm glass slides in 12‐well plates at 4000 cells per well and incubated with 5 ng·mL^−1^ TGF‐β or complete DMEM for 72 h. Cells were fixed with fixation buffer (2.5% glutaraldehyde solution, 4% formaldehyde in Sorensen's Phosphate Buffer, pH 7.4) for O/N at 4°C. The fixation buffer was discarded, and the slides were washed once with 1× DPBS and twice with dH_2_O for 10 min. Next, cells were fixed with 1% Osmium tetroxide (OsO_4_) at room temperature for 2 h and rinsed three times with dH_2_O for 10 min, followed by O/N incubation with 1% Uranyl acetate at 4°C. Afterwards, cells were gradually dehydrated using 50%, 70%, 90%, 96%, 100%, and 100% ethanol for 10 min at each step and incubated with the drying agent hexamethyldisilazane (HDMS) for O/N at room temperature. Coverslips were coated with gold nanoparticles and visualized with Zeiss Sigma500 FE‐SEM.

### 2.14 Zebrafish xenograft model

Zebrafish wild‐type AB +/+ strain was obtained from Izmir Biomedicine and Genome Center (IBG) Zebrafish Facility. Adult zebrafish were housed under standard conditions in an automated housing system (Zebtec CLS; Tecniplast, UK), at 28°C with a 14/10 h dark/light photoperiod; fish were fed twice daily with artemia and fish flakes (A1, B1, Otohime). Embryos were reared in embryo medium E3 (5 mm NaCl, 0.17 mm KCl, 0.33 mm CaCl_2_, 0.33 mm MgSO_4_, pH 7.2) at 28°C, in a petri dish at a density of 50 embryos/20 mL until 2 dpf. Animal experimentation was conducted in accordance with EU Directive 2010/63/EU, with permission from the IBG local ethics committee protocol number: 2024‐010. Xenograft injections were done to the yolk sac of 2 days post‐fertilization (dpf) embryos. Huh7 and Huh7‐TR cells were stained with DiI (V22885; Thermo Fisher Scientific, OR, USA) according to manufacturer directions; stained cells were suspended in 10% FBS prepared in PBS with a final concentration of 30 000 cells·μL^−1^. 150–200 cells were injected into each larva, and those with a local cell mass at the yolk were incubated at 34°C until 4 days post‐injection. If more than five cells exited the yolk, it was considered metastatic. The number of larvae with metastatic cells was calculated at 4 dpi in each group. Three replicates with 20–25 xenografts in each were quantified, and two independent experiments were conducted. The results of one representative experiment are presented.

### 2.15 Luciferase assay

TGF‐β/Smad signaling reporter constructs, pSBE4‐luc (Addgene plasmid #16495; Addgene, Watertown, MA, USA) or p3TP‐lux (Addgene plasmid #11767; Addgene, Watertown, MA, USA), were co‐transfected with pRL‐TK (Promega, Madison, WI, USA). Transfection experiments were carried out in biological replicates using Lipofectamine2000 (11668027; Thermo Fisher Scientific, Carlsbad, CA, USA) or PEI. Each transfection was performed in 24‐well plates and involved 200 ng of the reporter vector plus 3 ng of pRL‐TK. Post‐transfection (6 h), cells were supplemented with TGF‐β for 24 h. The luciferase reporter assay was performed using the Dual‐Glo Luciferase assay kit (E2920; Promega, Madison, WI, USA), as instructed by the manufacturer. The luminescence intensity of Firefly luciferase was normalized to Renilla luciferase.

### 2.16 Transcriptome analysis

RNA sequencing was carried out by a “stranded rRNA minus RNA‐seq” method on Illumina's NextSeq 500 platform at EMBL‐Genecore (Heidelberg, Germany). A minimum of 55 million 75 bp single‐end reads were generated per sample. For the computational analysis, low‐quality reads from the transcriptome libraries were removed using the “cutadapt ‐‐nextseq‐trim=20 ‐o {infile.fastq}” command with cutadapt v1.18 [25]. The remaining reads were analyzed using the human reference genome (GRCh38) and annotation data obtained from the Ensembl database (version 94). To measure gene expression levels, the rsem (v1.2.28) [26] index was built with the “rsem‐prepare‐reference ‐‐gtf {infile.gft} ‐‐bowtie2 {reference.fasta} {reference name}”' arguments, and subsequently, gene‐level expression data were measured from raw sequencing data using the “rsemcalculate‐expression ‐p {num} ‐‐output‐genome‐bam ‐‐bowtie2 {infile.fastq} {reference name} {outfile name}”' command and arguments. The statistical significance of differences in gene expression levels between groups was assessed in the r computing environment (v4.2.2) using the deseq2 package (v1.38.3) [27]. Count values determined by RSEM were transformed into a deseq2 object using the R‐package tximport, and subsequently, statistical significance of expression changes between groups was calculated using the DESeq() and results() functions. RSEM‐derived Transcript per Million (TPM) values were used for visualizing gene expression data. PCA analysis was performed using plotPCA function with default parameters from deseq2 package. The ggplot2 package in the R environment was used for visualizing expression data. Genes that showed differential expression were listed based on the false discovery rate (FDR). The gene list obtained from differential expression analysis was further dissected using the clusterprofiler (v4.6.2) or complexheatmap (v2.15.4) r packages [28, 29].

### 2.17 Gene set enrichment analysis

Gene set enrichment analysis (GSEA) was detailed previously [20]. Rather than scrutinizing individual gene expressions based on manually defined sets, this method assesses the significance of collective gene movements with common features. The “Huh7” “Huh7‐S” and “Huh7‐TR” groups were subjected to analysis for enriched gene sets using the GSEA tool provided by the Broad Institute. Briefly, a list of DEGs with phenotype labels was uploaded to the GSEA tool. Normalized enrichment scores (NES), nominal *P*‐values (*P*), and false discovery rates (FDR) were calculated based on 1000 random permutations of the gene set. bubble gum (GSEA Unlimited Map) software was used for calculations and graphical interface. NES > 1, *P* < 0.05, and an FDR < 0.25 were considered statistically significant, as specified in the user manual of the tool.

### 2.18 Statistical analysis

Results are presented as mean ± standard deviation (SD). Two‐tailed unpaired Student's *t*‐test was employed for experiments involving two experimental conditions or comparisons to assess differences between means. The calculations with *P*‐values < 0.05 were considered statistically significant. Detailed information on statistical significance is expressed in the figure legends. Statistical analyses were computed using graphpad prism 8.0 (GraphPad Software, San Diego, CA, USA).

## 3 Results

### 3.1 Huh7 cell line is a unique model for TGF‐β‐induced senescence

To bring forward and introduce the Huh7 cell line as a relevant model for investigating the cellular and molecular aspects of TGF‐β‐induced senescence and the acquisition of an optimal proliferation rate in resilient cells, we performed a series of experiments. Initially, cells were stimulated with TGF‐β at near physiological levels (5 ng·mL^−1^). As early as 3 days post‐treatment, cells displayed significant growth suppression coupled with key phenotypic and functional hallmarks of senescent cells. These included dynamic, time‐dependent alterations in well‐known senescence markers, including p‐pRb (S807/811), p15, and p21 (Fig. S1A), flattened and enlarged morphology (Fig. S1B,C), and increased SA‐β‐Gal activity (Fig. S1D). Corroborating the specificity of senescence entry, we observed a strong reduction in BrdU incorporation, with a significant proportion of the cell population losing its ability to synthesize new DNA (Fig. S1E). Further characterization revealed abnormal nuclear morphology of senescent cells, as shown by Lamin A/C and DAPI staining (Fig. S1F). Antiproliferative effects manifested by TGF‐β were linked to cell cycle inhibition, as evidenced by G1 arrest and a strong reduction in S‐phase cells (Fig. S1G). These phenotypes coincided with a substantial decrease in the colony formation ability of Huh7 cells (Fig. S1H).

### 3.2 Smad3 plays a dominant role in senescence induction

**Fig. 1:**
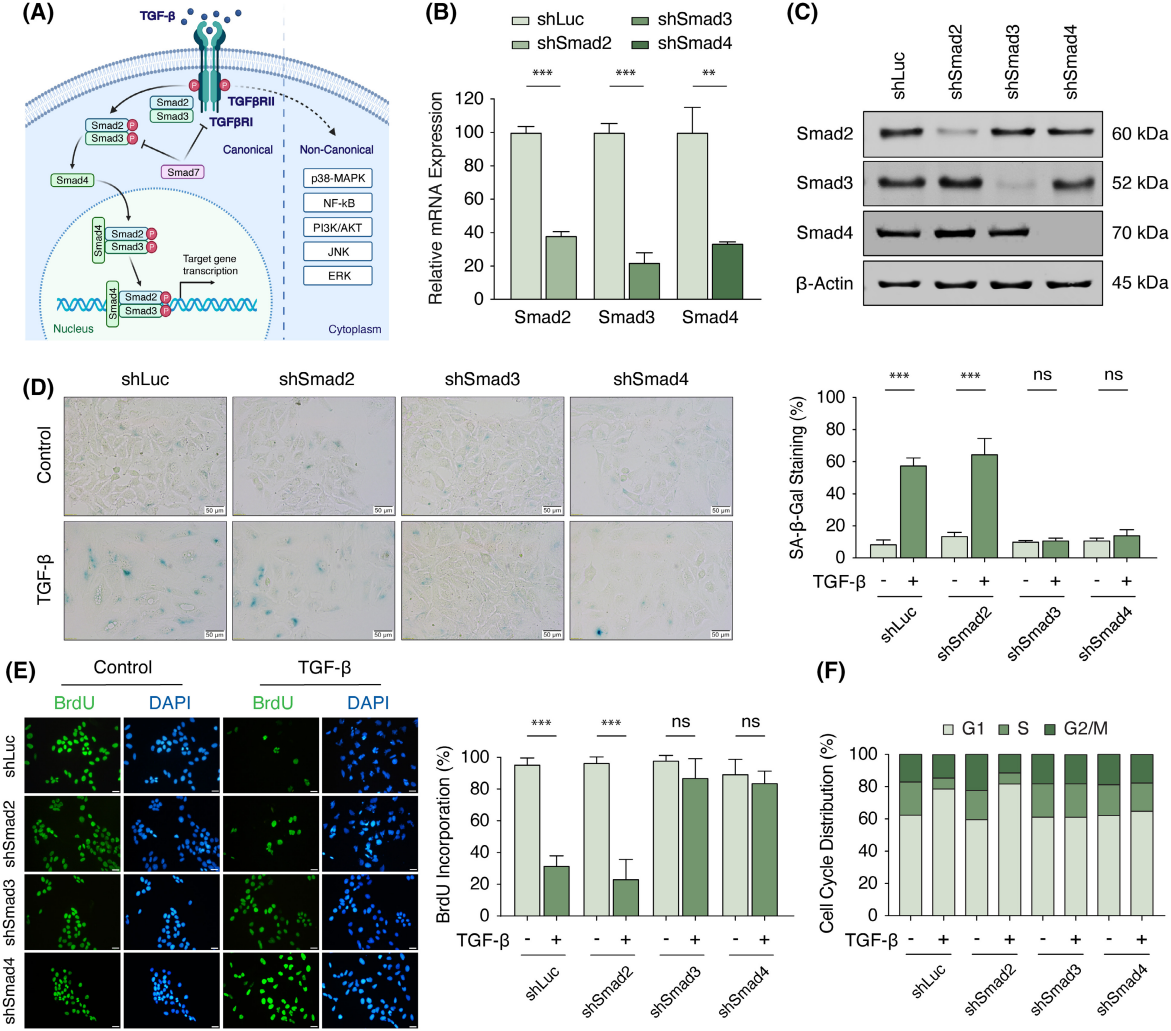
TGF‐β‐induced cellular senescence is mediated through Smad3 signaling. (A) Schematic diagram of the canonical and non‐canonical TGF‐β signaling pathways, created with BioRender.com. (B) qRT‐PCR confirmation for successful depletion of Smad molecules upon shRNA knockdown. Levels of Smad gene expression were normalized against the expression of the housekeeping gene, β‐Actin, using the $2^{\Delta \Delta C_{t}}$ formula. shLuc against firefly luciferase mRNA served as a non‐human targeting control. (C) Knockdown validation of Smad molecules via western blotting. β‐Actin served as the loading control. (D) Suppression of the TGF‐β senescence response in shSmad3 and shSmad4 clones. SA‐β‐Gal activity was measured in cells left untreated or treated with 72 h of TGF‐β. Scale bar: 50 μm. (E) TGF‐β treatment (72 h) exerts no effect on BrdU incorporation in Smad3 and Smad4 knockdown clones. Scale bar: 20 μm. (F) Cell cycle analysis in Smad knockdown clones after 72 h TGF‐β treatment was performed using PI staining. Relevant data are plotted as mean ± SD on three replicates. All statistical analyses were performed with two‐tailed Student's *t*‐test. *P*‐values, TGF‐β (5 ng·mL^−1^, 72 h) versus control. **P < 0.01; ***P < 0.001; ns, not significant. BrdU, bromodeoxyuridine; PI, propidium iodide; qRT‐PCR, quantitative reverse transcription polymerase chain reaction; SA‐β‐Gal, senescence‐associated β‐galactosidase; shLuc, short hairpin luciferase; TGF‐β, transforming growth factor‐β.

To probe the mechanism of senescence induction by canonical TGF‐β/Smad signaling (Fig. 1A), we suppressed the expression of Smad genes using retroviral RNAi vectors (Fig. 1B,C). Silencing Smad3 or the common mediator Smad4, but not Smad2, abolished hallmark senescence features, including SA‐β‐Gal activity (Fig. 1D) and flattened cell morphology (Fig. S2A). Additionally, the loss of Smad3 or Smad4 mitigated TGF‐β‐mediated abnormal nuclear shape (Fig. 1E, DAPI staining), as well as the suppression of DNA synthesis (Fig. 1E) and G1 cell cycle arrest (Fig. 1F). These findings suggest that Smad3, along with Smad4, plays a dominant role in manifesting TGF‐β‐induced growth arrest and cellular senescence in Huh7 cells.

### 3.3 Chronic TGF‐β exposure promotes clonal regrowth and induces TGF‐β resistance

**Fig. 2:**
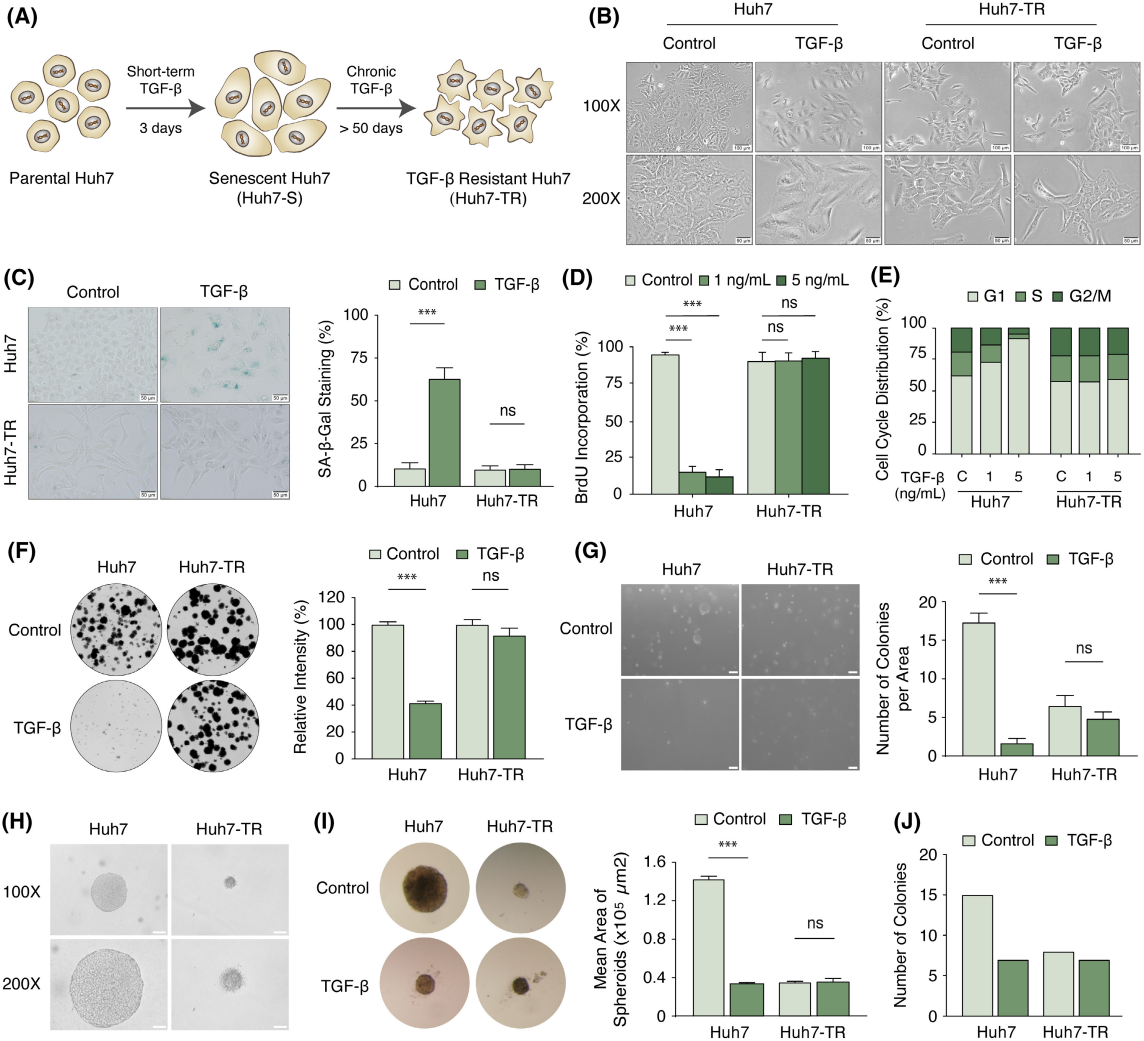
Chronic TGF‐β exposure induces senescence escape and TGF‐β resistance. (A) Schematic diagram of short‐term and long‐term TGF‐β treatment. The scheme was created with BioRender.com. (B) Morphological changes in Huh7 and Huh7‐TR cells after 72 h of TGF‐β (5 ng·mL^−1^) treatment. Upper panel: 100×, scale bar: 100 μm; lower panel: 200×, scale bar: 50 μm. (C) Representative images of the SA‐β‐Gal assay in Huh7 and Huh7‐TR cells after 72 h of TGF‐β (5 ng·mL^−1^) treatment. Scale bar: 50 μm. Bar graphs are presented as the mean ± SD of three replicates. (D) Resistance to inhibition of BrdU incorporation in Huh7‐TR cells. Cells were treated with 1 and 5 ng·mL^−1^ TGF‐β for 72 h. Cells positively labeled for BrdU (24 h) were stained with immunofluorescence technique using anti‐BrdU (mouse) primary antibody, which was followed by anti‐mouse Alexa‐488 secondary antibody incubation. Eight areas were counted in each triplicate assay (Control: no TGF‐β treatment). (E) Cell cycle analysis was performed using PI staining in Huh7 and Huh7‐TR cells after dose‐dependent TGF‐β treatment. C, control. (F) 2D colony formation capacity of Huh7 and Huh7‐TR cells in the absence and presence of 5 ng·mL^−1^ TGF‐β. The assay was performed in triplicates in 6‐well cell culture plates for 25 days, followed by crystal violet staining. High‐resolution images were captured with the LI‐COR Odyssey CLx Imaging System, and the relative crystal violet intensities were quantified using image studio lite image processing software. (G) 3D soft agar anchorage‐independent growth capacity of Huh7 and Huh7‐TR cells in the absence and presence of 5 ng·mL^−1^ TGF‐β. The assay was performed in triplicates in 12‐well cell culture plates. The colonies were measured using imagej software. The 5000 μm^2^ colony area in the control group was accepted as the threshold, and the colonies with an area larger than this value were counted in all groups. Scale bar: 200 μm. (H) Representative images showing reduced 3D soft agar anchorage‐independent growth of Huh7‐TR cells. Images were obtained on an Olympus IX71 microscope. Upper panel: 100×, scale bar: 100 μm; lower panel: 200×, scale bar: 50 μm. (I) Representative images of spheroids in the hanging droplet assay. TGF‐β treatment reduced the 3D spheroid‐forming potential of Huh7 cells, whereas Huh7‐TR cells were unaffected. Spheroids were imaged using stereo microscopy (6.3× zoom). The assays were performed in three biological replicates with 20 technical repeats. The mean area of spheroids (μm^2^) was calculated using imagej. (J) The 34 000 μm^2^ colony area in the control group was accepted as the threshold, and the colonies with an area larger than this value were quantified in all groups. All data in bar charts are plotted as mean ± SD of three replicates, C or Control: no TGF‐β treatment. All statistical analyses were computed with a two‐tailed Student's *t*‐test. *P*‐values, TGF‐β versus control; ***P < 0.001; ns, not significant. 2D, two‐dimensional; 3D, three‐dimensional; BrdU, bromodeoxyuridine; Huh7‐TR, TGF‐β resistant; PI, propidium iodide; SA‐β‐Gal, senescence‐associated β‐galactosidase; TGF‐β, transforming growth factor‐β.

Having elucidated the robust senescence response by short‐term TGF‐β stimulation, we explored how Huh7 cells react to chronic TGF‐β exposure. Continuous monitoring of senescent cells enriched by FACS‐sorting for high SA‐β‐gal activity and senescence marker expression using C12FDG (Fig. S2B,C), a lipophilic green fluorescent substrate for β‐galactosidase [24], suggested that these cells remained in an arrested state for approximately 3–4 weeks into TGF‐β treatment. At this juncture, subpopulations of Huh7 cells began to show clonal expansion with the acquisition of an optimal division rate (Fig. S2B). Chronic TGF‐β exposure ultimately culminated in the emergence of a TGF‐β resistant polyclonal subline, which we labeled Huh7‐TR (Fig. 2A). Upon immediate comparison to parental cells, Huh7‐TR cells demonstrated a distinctive phenotype, marked by a lack of response to TGF‐β‐mediated morphologic alterations (Fig. 2B), senescence induction (Fig. 2C), inhibition of DNA synthesis (Fig. 2D), and G1 cell cycle arrest (Fig. 2E and Fig. S3A). Further monitoring of cell cycle progression using the fluorescence ubiquitination‐based cell cycle indicator (FUCCI) reporter [30] (Fig. S3B) confirmed differential TGF‐β responses in Huh7 and Huh7‐TR cell models (Fig. S3C). Remarkably, Huh7‐TR cells remained susceptible to cell cycle arrest and senescence induction by doxorubicin treatment (Doxo), emphasizing the TGF‐β selective nature of the resistance phenotype (Fig. S3D–F).

The TGF‐β resistant state proved to be permanent, as Huh7‐TR cells did not revert to a sensitive phenotype upon TGF‐β withdrawal. This suggested that the acquisition of TGF‐β resistance might stem from the disruption or inactivation of the signaling cascade. To test this hypothesis, we assayed for pathway responses using TGF‐β reporters, pSBE4‐luc and p3TP‐lux [19]. These efforts revealed a strong attenuation of transcriptional activation in Huh7‐TR cells (Fig. S4A,B). In line with these results, we found that transcriptional and functional activation of senescence‐related genes was absent in Huh7‐TR cells at both the transcript (Fig. S4C–F) and protein levels (Fig. S4G). We next interrogated whether the loss of senescence‐related phenotypes was concomitant with changes in the cellular and functional features of aggressive cancer cells. Under 2D low‐clonogenicity conditions, Huh7‐TR cells exhibited a greater number and larger size of colonies compared to untreated Huh7 cells, with no effect exerted by TGF‐β treatment (Fig. 2F). Interestingly, under 3D anchorage‐independent soft agar conditions, resistant cells showed reduced spheroid formation relative to untreated parental cells (Fig. 2G). A closer examination of spheroid borders in untreated Huh7‐TR cells revealed spindle‐shaped protrusions, an indication of mesenchymal invaginating cells (Fig. 2H). We also examined the ability of cells to spontaneously aggregate into spheroids in hanging‐drop cultures. As shown in Fig. 2I,J, spheroids derived from Huh7‐TR cells displayed a significantly smaller area, formed fewer colonies, and, unlike Huh7 cells, remained unresponsive to TGF‐β treatment.

To evaluate the broader applicability of this phenomenon, we investigated Hep3B as an additional epithelial HCC cell line with a p53‐null and RB1‐deficient background [31, 32]. Our results indicated that chronic TGF‐β exposure can similarly induce resistance in other HCC cell lines that initially show a senescence response. The newly established TGF‐β resistant model, Hep3B‐TR, also demonstrated phenotypic transition from an epithelial‐like appearance to an elongated, fibroblast‐like morphology (Fig. S5A,F), and displayed insensitivity to TGF‐β rechallenge, failing to undergo the expected senescence or growth arrest or activate transcriptional responses (Fig. S5B–E). Consistent with earlier reports that have linked chronic TGF‐β exposure to the acquisition of mesenchymal stem‐related traits in epithelial Hep3B and PLC/PRF/5 HCC cell lines [33], our results indicate that Huh7 or Hep3B subpopulations resuming proliferation following TGF‐β‐induced senescence may eventually acquire unique hallmarks associated with the functional and phenotypic plasticity of tumor cells.

### 3.4 Clonal regrowth is associated with the adoption of mesenchymal features

**Fig. 3:**
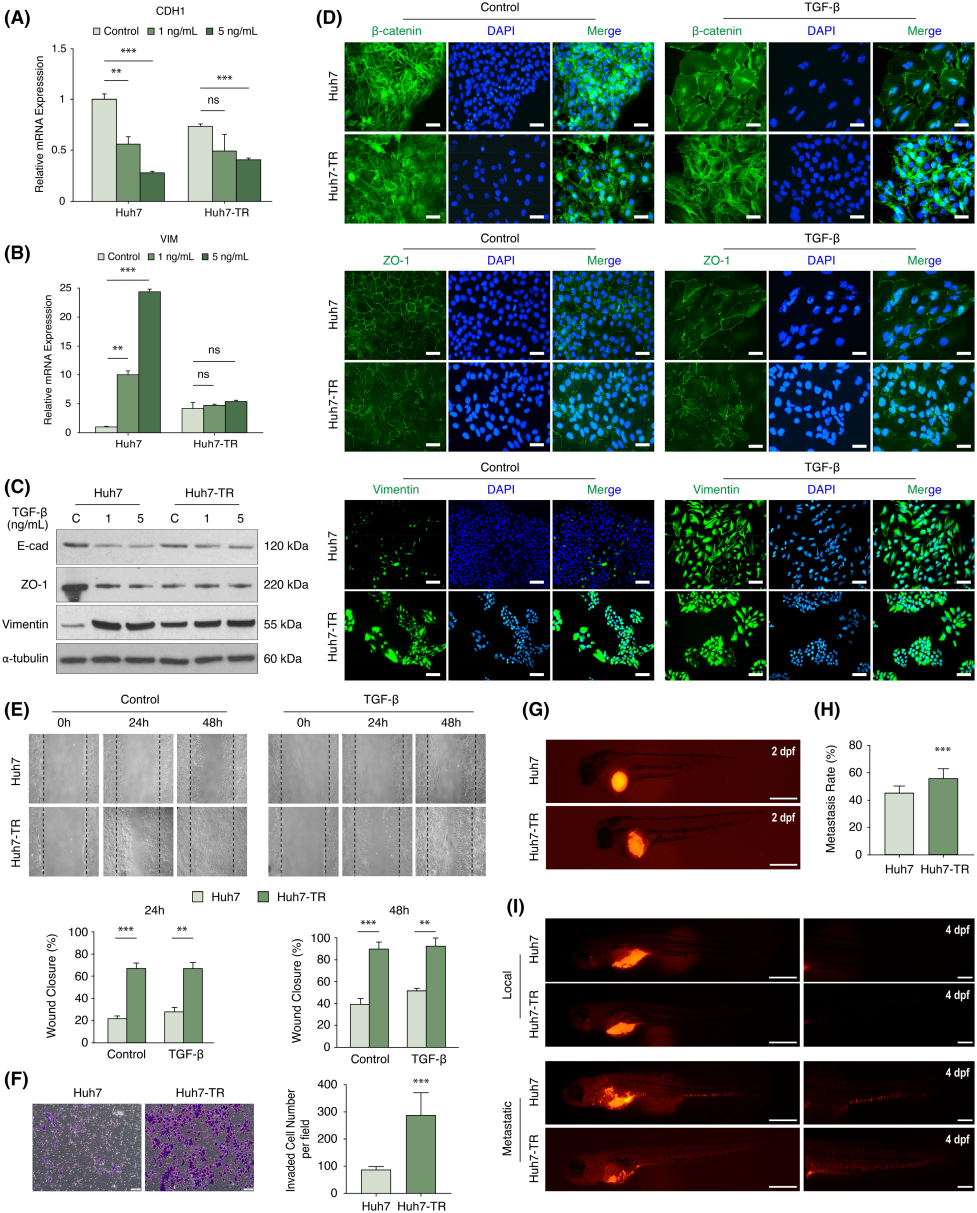
Evasion from TGF‐β‐induced senescence is associated with a mixed EMT phenotype. (A, B) Transcript levels of epithelial (CDH1) and mesenchymal (VIM) markers in Huh7 and Huh7‐TR cells. Cells were treated with 1 or 5 ng·mL^−1^ TGF‐β for 72 h. qRT‐PCR analysis was performed with gene‐specific primer pairs. Expression values were normalized with respect to GAPDH. (C) Protein expression of E‐cadherin, ZO‐1, and Vimentin in Huh7 and Huh7‐TR cells following 1 or 5 ng·mL^−1^ TGF‐β treatment for 72 h. Western blotting analysis was performed with corresponding primary antibodies. α‐tubulin was monitored as equal loading. (D) Staining patterns of β‐catenin, ZO‐1, and Vimentin in Huh7 and Huh7‐TR cells. Scale bar: 50 μm for β‐catenin and ZO‐1, and 100 μm for Vimentin. (E) Differential motility of Huh7 and Huh7‐TR cells in the absence and presence of TGF‐β (5 ng·mL^−1^). Wound closure capacity was measured at 24 and 48 h. Cells were cultured in 12‐well culture plates, and the linear wounds were created with a pipette tip in confluent monolayer cells. Phase‐contrast pictures were obtained at ×20 magnification. Dashed lines indicate initial edges after scratching at 0 h. At least five measurements were used in the calculation of distances between wound edges. (F) Transwell invasion assay in Huh7 and Huh7‐TR cells. Scale bar: 100 μm. Invaded cell numbers per field (*n* = 11) were calculated by manual counting using imagej software. (G) Huh7 and Huh7‐TR cells were injected into 2 dpf zebrafish yolk; representative images are displayed. Scale bar: 500 μm. (H) Metastatic rates of Huh7 (44.66%) and Huh7‐TR (55.81%) injected groups at 4 dpi. (I) Metastasis analysis was conducted at 4 dpi. Images are representative of all groups. Scale bar: left panel, 500 μm; right panel, 200 μm. All data in bar charts are presented as the mean ± SD from three replicates. C or Control: no TGF‐β treatment. Statistical significance was calculated by a two‐tailed Student's *t*‐test. **P < 0.01; ***P < 0.001; ns, not significant. CDH1, E‐cadherin; dpf, days post fertilization; dpi, days post injection; EMT, epithelial‐to‐mesenchymal transition; Huh7‐TR, TGF‐β resistant; qRT‐PCR, quantitative reverse transcription polymerase chain reaction; TGF‐β, transforming growth factor‐β; VIM, vimentin; ZO‐1, zonula occludens‐1.

TGF‐β is widely known to induce epithelial‐to‐mesenchymal transition (EMT) in HCC cells as part of its pro‐tumorigenic functions [33]. This process entails the disruption of polarized epithelial morphology and transformation into spindle‐shaped cells with reduced adhesion at cellular junctions. To investigate whether the distinctive features observed in Huh7‐TR cells could be attributed to EMT, we assessed the expression levels and subcellular localization of well‐established epithelial and mesenchymal markers. Comparative analysis with parental Huh7 cells revealed a reduction, but not a complete loss, in the transcript levels of CDH1 (encoding E‐cadherin) and a significant upregulation of VIM (encoding Vimentin), which could be replicated at the protein level (Fig. 3A–C). Additionally, Huh7‐TR cells showed a spindle‐shaped phenotype characterized by a breakdown in epithelial cell integrity (β‐catenin), dissolution of tight junctions (Zonula occludens‐1, ZO‐1), and reorganization of intracellular filamentous structures (Vimentin) (Fig. 3D). Consistent with their mesenchymal‐like nature, resistant cells demonstrated enhanced migration (Fig. 3E), increased Matrigel invasion (Fig. 3F), and higher metastatic potential in zebrafish xenograft models (Fig. 3G–I). These findings corroborate prior reports suggesting that mesenchymal HCC cells have a greater capacity for motility and invasion [34]. Critically, unlike the reversible TGF‐β‐induced EMT seen in normal mammary epithelial cells, which enables escape from TGF‐β cytostasis [35], forced induction of a mesenchymal‐like state via E‐cadherin silencing did not confer TGF‐β resistance in Huh7 cells (Fig. S6A–D). This suggests that EMT alone may not be an immediate prerequisite or sufficient to counteract TGF‐β cytostasis in epithelial HCC cells. Furthermore, contrary to our expectations of an association between EMT plasticity and stemness [36], the mixed epithelial‐mesenchymal phenotype of Huh7‐TR cells did not correlate with changes in the expression or localization of key HCC stemness markers, such as EpCAM, CD44, CD90, CD133, and CK19 (Fig. S7A,B). This suggests that chronic TGF‐β treatment does not selectively enrich the pre‐existing cancer stem cell population in Huh7 cells [37].

### 3.5 Loss of TGF‐β sensitivity is associated with defective TGF‐β/Smad signaling

**Fig. 4:**
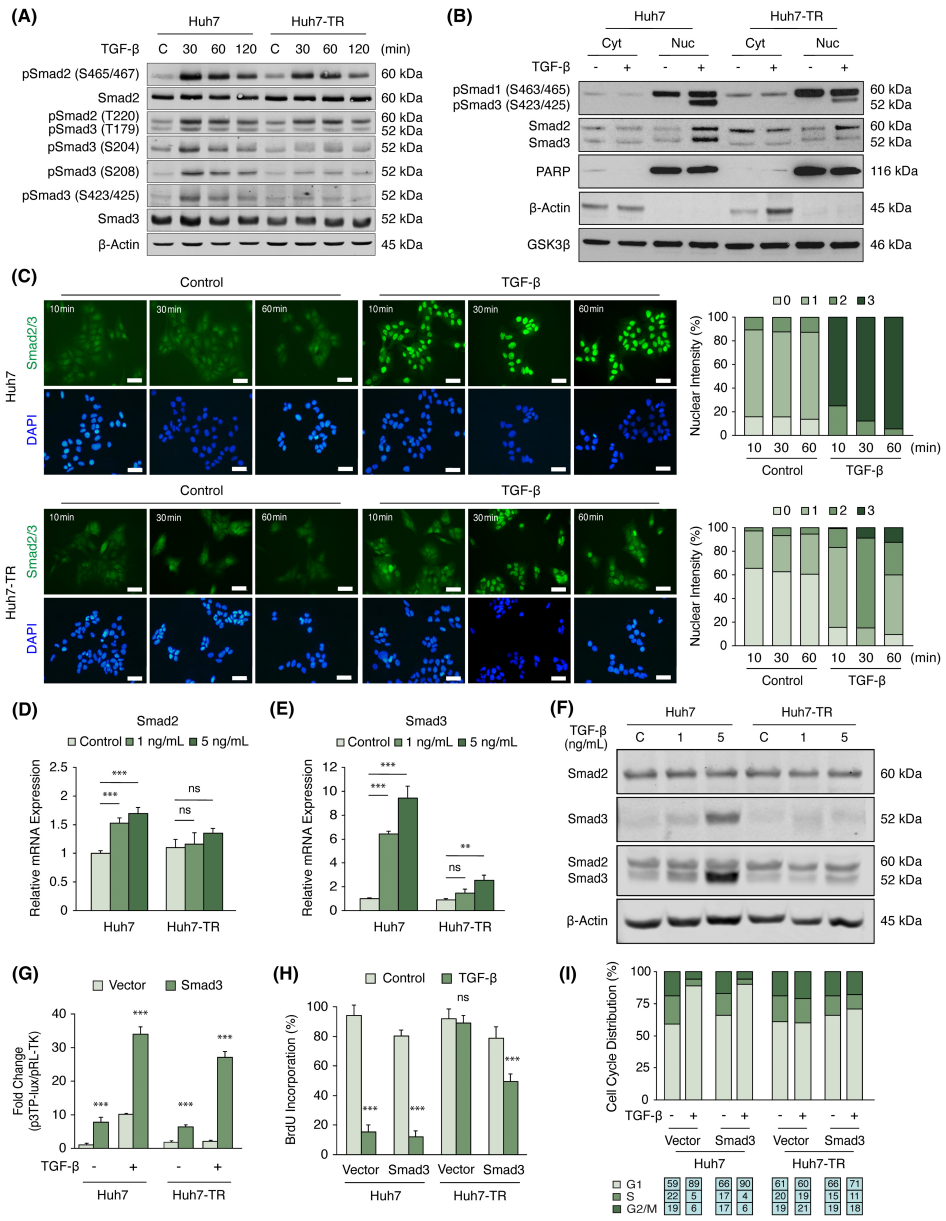
TGF‐β/Smad signaling axis is defective in Huh7‐TR cells. (A) Time‐resolved protein expression of Smad2 and Smad3 in Huh7 and Huh7‐TR. β‐Actin served as the loading control. (m, minute) (B) Nuclear/cytoplasmic shuttling of Smad molecules, particularly Smad3, is impaired in Huh7‐TR cells. Cells were treated with 5 ng·mL^−1^ TGF‐β for 60 min. Cytoplasmic and nuclear fractions were obtained accordingly. Western blotting analysis was performed with corresponding primary antibodies. PARP and β‐Actin were used as controls for nuclear and cytoplasmic fractions, respectively. GSK3β was used as a control for equal loading. (C) Decreased nuclear accumulation and time‐dependent delay in Smad2/3 translocation in Huh7‐TR. Cells were treated with 5 ng·mL^−1^ TGF‐β for specified time periods. Scale bar: 20 μm. (D, E) Transcript levels of Smad2 and Smad3. Huh7 and Huh7‐TR cells were treated with 1 and 5 ng·mL^−1^ TGF‐β for 72 h. qRT‐PCR analysis was performed with corresponding primer pairs. Expression values were normalized to GAPDH. (F) Protein expression of Smad2 and Smad3 in Huh7 and Huh7‐TR. Cells were treated with 1 and 5 ng·mL^−1^ TGF‐β for 72 h. Western blotting analysis was performed with corresponding primary antibodies, either with Smad2 and Smad3 alone or with Smad2/3 antibodies. β‐Actin was monitored as equal loading. (G) Ectopic Smad3 expression restores TGF‐β responses in Huh7‐TR cells. Cells were co‐transfected either with pcDNA3.1 empty backbone or pcDNA3.1‐Smad3‐WT, and p3TP‐Lux and pRL‐TK plasmids. Transfected cells were enriched by antibiotic selection for 3 days and then left untreated or treated with TGF‐β (5 ng·mL^−1^) for 24 h. The luciferase activity was measured and expressed as fold change of Reporter/pRL‐TK. (H) BrdU incorporation in Huh7 and Huh7‐TR after TGF‐β treatment following ectopic Smad3 expression. Following transfection (G), cells were treated with TGF‐β (5 ng·mL^−1^) for 72 h. Percent BrdU was calculated by manual counting of at 6 areas from each triplicate experiment. (I) Cell cycle analysis in Huh7 and Huh7‐TR after 72 h TGF‐β (5 ng·mL^−1^) treatment following ectopic Smad3 expression. All data in bar charts are presented as the mean ± SD from three replicates. C or Control: no TGF‐β treatment. Statistical significance was calculated by a two‐tailed Student's *t*‐test. **P < 0.01; ***P < 0.001; ns, not significant. BrdU, bromodeoxyuridine; h, hour; Huh7‐TR, TGF‐β resistant; qRT‐PCR, quantitative reverse transcription polymerase chain reaction; TGF‐β, transforming growth factor‐β.

To dissect the molecular cues underpinning resistance to TGF‐β, we analyzed the integrity of TGF‐β/Smad signaling in Huh7‐TR cells. Our initial findings upon acute TGF‐β stimulation revealed reduced phosphorylation of Smad3 at C‐terminal residues (S423/S425), while Smad2 activation (S465/467) remained largely intact with only a mild inhibition. Moreover, phosphorylation of Smad3 at linker residues (T179, S204, S208), a modification known to attenuate tumor‐suppressive functions of Smad3 [38], was also significantly diminished (Fig. 4A). These pathway modifications persisted under prolonged TGF‐β stimulations (Fig. S8A). Under both experimental conditions, basal levels of Smad3 linker phosphorylation were comparable between parental and Huh7‐TR cells, negating the intrinsic engagement of this mechanism in TGF‐β insensitivity. Subsequent exploration into Smad3 functionality unfolded a defect in nuclear accumulation of both phosphorylated and total Smad3 in Huh7‐TR cells (Fig. 4B and Fig. S8B,C). Time‐resolved analysis of signaling kinetics further substantiated this aberration, demonstrating impaired trafficking characterized by delayed nuclear import or reduced nuclear retention of Smad molecules, Smad2/3 and Smad4 (Fig. 4C and Fig. S9). Another critical aspect of senescence induction in Huh7 cells was the TGF‐β‐dependent upregulation of Smad3, but not Smad2 (Fig. 4D–F), corroborating the selective impairment of this molecule in Huh7‐TR cells. Hence, ectopic expression of Smad3 was able to reinstate TGF‐β sensitivity, restoring transcriptional responses (Fig. 4G), suppressing BrdU incorporation (Fig. 4H), and inducing G1 cell cycle arrest (Fig. 4I). Despite a notable increase in SA‐β‐Gal activity, Smad3 overexpression did not manifest enlarged cell morphology even after 5 days of TGF‐β treatment (Fig. S10A–C). In‐depth examination of signaling components in Huh7‐TR cells unveiled diminished expression of TGF‐β receptors, particularly TGFβRI (Fig. S10D). Consistently, the stable overexpression of TGFβRI resulted in a modest TGF‐β resensitization. This was evidenced by an elevation in SA‐β‐Gal activity with a reciprocal reduction in BrdU incorporation, and only a slight increase in G1 cell cycle arrest (Fig. S10E–H). Finally, a marked upregulation in the baseline expression of TGF‐β1 ligand in Huh7‐TR cells indicated a potential reinforcement of TGF‐β selective pressure to maintain insensitivity (Fig. S10I). Collectively, these data establish a link between TGF‐β insensitivity and defective Smad signaling in Huh7‐TR cells.

### 3.6 Senescent and resistant cells have distinct transcriptomic landscapes

**Fig. 5:**
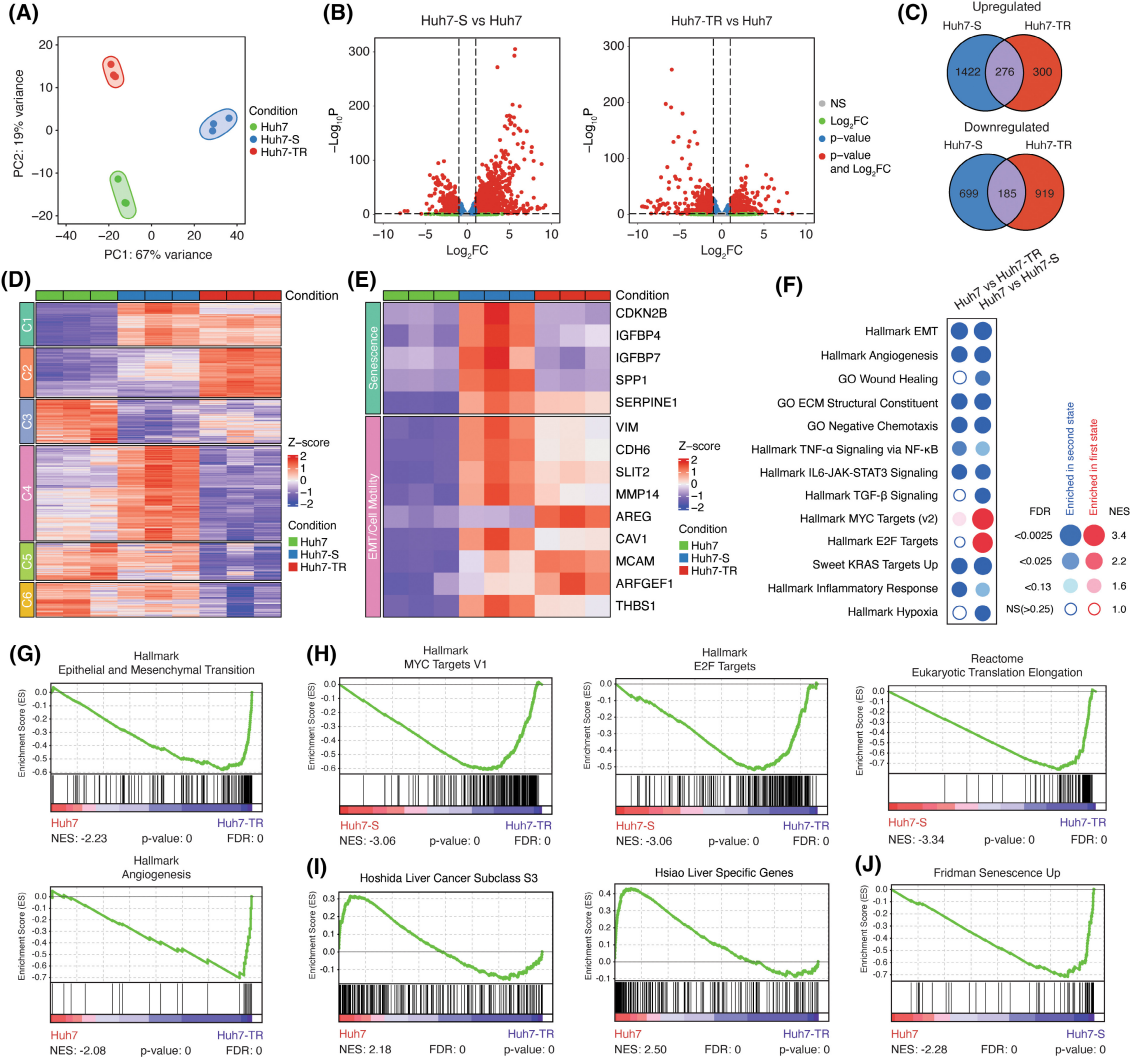
Transcriptomic landscape of TGF‐β sensitive, senescent, and resistant states. (A) Principal component analysis of RNA‐Seq data for three states. (B) Volcano plot showing the distribution of differentially expressed genes between Huh7 vs Huh‐S (left) as well as Huh7 vs Huh7‐TR (right) states. Red dots display significantly differentially expressed genes, meeting both *P*‐value < 0.05 and Log_2_FC ≥ 1 or ≤ −1 criteria. Blue dots display genes that only meet *P*‐value < 0.05 criteria. Green dots display genes that meet Log_2_FC ≥ 1 or ≤ −1 criteria but are not statistically significant *P*‐value > 0.05. Gray dots display genes that are not significantly differentially expressed. (C) The number of differentially upregulated and downregulated, shared or not shared, genes between senescent (Huh7‐S) and resistant (Huh7‐TR) states (*P*‐value < 0.05, Log_2_FC ≥ 1 and Log_2_FC ≤ −1). (D) Clustered heatmap of gene expression changes with similar or unique trends. The top 2000 genes (ranked by FDR) identified to change significantly in Huh7‐TR cells in any experimental comparison using a moderated *F*‐statistic were listed. Gene expression values were *Z*‐score normalized, and patterns were identified by *K*‐means clustering (*K* = 6) and subclustered by Euclidean distance. (E) Heatmap demonstrating the expression changes of senescence‐ and EMT‐related genes in three different states (condition). (F) BubbleGUM visualization of GSEA using MSigDB, including Hallmark, Gene Ontology, and curated (C2) gene sets. Normalized enrichment score (NES) ≥ 1, FDR value: ≤ 0.25. (G–J) GSEA of select gene signatures based on differentially expressed genes between different states according to the RNA‐seq data. ES represents enrichment score. EMT, epithelial‐to‐mesenchymal transition; FDR, false discovery rate; GSEA, gene set enrichment analysis; Huh7‐S, TGF‐β‐induced senescence; Huh7‐TR, TGF‐β resistant; MSigDB, The Molecular Signatures Database.

To define the gene expression profiles of TGF‐β sensitive and resistant states, we performed an integrative RNA‐seq analysis of three conditions: (a) parental Huh7 cells, (b) Huh7 cells undergoing TGF‐β‐induced senescence (Huh7‐S), and (c) Huh7‐TR cells. Principal component analysis (PCA) revealed distinct transcriptomic landscapes between each state (Fig. 5A and Fig. S11A). Differential gene expression analyses enabled the identification of significantly upregulated and downregulated genes (Fig. 5B), many of which were not shared between senescent and resistant states (Fig. 5C and Fig. S11B). Cluster profiling of the most differentially expressed genes revealed alterations characterized by either similar or distinctive trends. We found several interesting patterns, such as cluster 2, which contained genes selectively upregulated in Huh7‐TR cells compared to parental and senescent cells (Fig. 5D and Table S4). Cluster 4, on the other hand, contained genes that were upregulated in the senescent state (Huh7‐S vs Huh7), yet downregulated upon evolving into the resistant state (Huh7‐TR vs Huh7‐S). Cluster 5 and cluster 6 were enriched for genes that were uniquely downregulated in Huh7‐TR cells, with no significant alterations between Huh7 and Huh7‐S cells (Fig. 5D). Among the genes upregulated in Huh7‐S cells were those that have been shown to probe senescence‐like phenotypes, such as CDKN2B [12], IGFBP4 and IGFBP7 [39], SPP1 [40], and SERPINE1 [41]. Conversely, Huh7‐TR cells exhibited elevated expression of TGF‐β targets associated with EMT (VIM, CDH6), cell migration (SLIT2, MMP14, AREG, and CAV1), and wound healing (MCAM, ARFGEF1, and THBS1) (Fig. 5E). Consistently, gene set enrichment analysis (GSEA) reinforced EMT and angiogenesis as hallmarks of the resistance phenotype (Fig. 5F,G). The enrichment of extracellular matrix (ECM) constituents and core matrisome gene signatures further emphasized the motile and invasive nature of Huh7‐TR cells (Fig. S11C). These findings align with studies showing that EMT and ECM remodeling can promote cancer cell dissemination [42]. In line with gene expression changes (Fig. 5E), the EMT gene signature showed modest enrichment in Huh7‐S cells (Fig. 5F), suggesting that EMT and senescence phenotypes are not mutually exclusive in the senescent state. Pathways such as IL6/JAK/STAT3, TNF‐α/NF‐kB, and inflammatory response were upregulated in both states, but TGF‐β signaling was enriched in the senescent state (Fig. 5F and Fig. S11D). The enrichment for eukaryotic translation, MYC, and E2F target gene expression supported the proliferative capacity of Huh7‐TR cells relative to Huh7‐S cells (Fig. 5H). Gene signatures associated with well‐differentiated HCC were more prevalent in Huh7 cells, supporting our previous conclusions that a less‐differentiated phenotype is induced in Huh7‐TR cells (Fig. 5I). Furthermore, we identified a subset of senescence‐related gene sets (i.e., Senescence, RB1, and KRAS) that were differentially regulated in Huh7‐S cells (Fig. 5J and Fig. S11E). Finally, we found reduced mitochondrial gene expression in Huh7‐S cells (Fig. S11F), suggesting potential metabolic alterations associated with the senescent state [43]. Together, cluster profiling and GSEA data support our earlier conclusions, indicating that the selection of the TGF‐β resistant state resulted in distinct gene regulatory programs that differ from senescent and parental cells.

### 3.7 Novel genes modulating TGF‐β responses

**Fig. 6:**
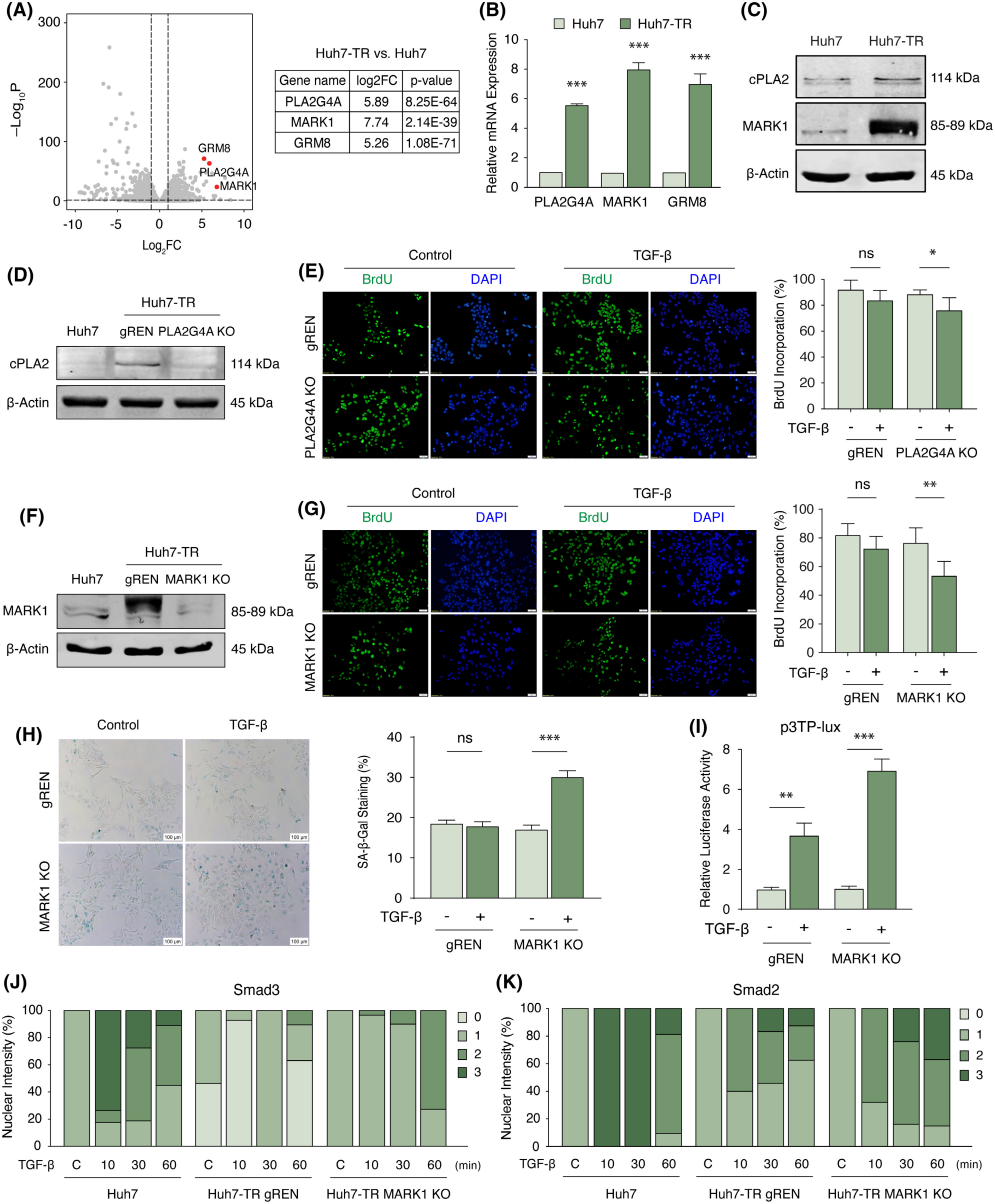
Transcriptome profiling identifies differentially expressed genes associated with resistance to TGF‐β. (A) Volcano plot representation of differentially expressed genes between Huh7‐TR vs Huh7 states. PLA2G4A, MARK1, and GRM8 genes are labeled with red dots. (B) Transcript levels of PLA2G4A, MARK1, and GRM8 genes in Huh7‐TR cells. qRT‐PCR analysis was performed with gene‐specific primer pairs. Expression values were normalized with β‐Actin. (C) Validation of PLA2G4A (cPLA2) and MARK1 protein levels in Huh7‐TR cells. (D) Western blot confirmation of cPLA2 genetic depletion (KO) through CRISPR/Cas9 targeting in Huh7‐TR cells. Cells were infected with pECPV‐Renilla (control) or pECPV‐PLA2G4A vectors and enriched by cell sorting against Venus expression. Huh7 cells were used as a control. (E) BrdU incorporation in Huh7‐TR cells after TGF‐β treatment following depletion of PLA2G4A expression. Cells were treated with TGF‐β (5 ng·mL^−1^) for 72 h. Percent BrdU was calculated by manual counting of 8 areas from each triplicate experiment. Scale bar: 50 μm. (F) Western blot confirmation of MARK1 knockout (KO) through CRISPR/Cas9 targeting in Huh7‐TR. Cells were infected with pECPV‐Renilla (control) or pECPV‐MARK1 vectors and enriched by cell sorting against Venus expression. Huh7 cells were used as a control. Western blotting analysis was performed with corresponding primary antibodies. β‐Actin served as the equal loading control. (G) BrdU incorporation in Huh7‐TR cells after TGF‐β treatment following depletion of MARK1 expression. Cells were treated with TGF‐β (5 ng·mL^−1^) for 5 days. Percent BrdU was calculated by manual counting of 8 areas from each triplicate experiment. Scale bar: 50 μm. (H) SA‐β‐Gal activity identified increased cytostatic responses to TGF‐β activity in MARK1 knockout cells compared to control cells (gREN). Representative images are shown (SA‐β‐Gal staining in blue), scale bar: 100 μm. (I) MARK1 attenuates TGF‐β/Smad3 transcriptional activity. Cells stably transduced either with pECPV‐Renilla (control) or pECPV‐MARK1 were co‐transfected with p3TP‐Lux and pRL‐TK plasmids and then left untreated or treated with TGF‐β (5 ng·mL^−1^) for 24 h. The luciferase activity was measured and expressed as fold change of Reporter/pRL‐TK. (J, K) MARK1 dysregulates time‐resolved signaling dynamics of nuclear Smad3 and Smad2. Huh7 cells were used as a control. All data in bar charts are presented as the mean ± SD from three replicates. Statistical significance was calculated by a two‐tailed Student's *t*‐test. *P < 0.05, **P < 0.01, and ***P < 0.001; ns, not significant. BrdU, bromodeoxyuridine; GRM8, glutamate metabotropic receptor 8; Huh7‐TR, TGF‐β resistant; MARK1, microtubule affinity regulating kinase 1; MARK1, microtubule affinity regulating kinase 1; PLA2G4A, phospholipase A2 group IVA; qRT‐PCR, quantitative reverse transcription polymerase chain reaction; SA‐β‐Gal, senescence‐associated β‐galactosidase; TGF‐β, transforming growth factor‐β.

Given the substantial differences in gene expression patterns between TGF‐β sensitive and insensitive cells, we reasoned that multiple genes may concurrently contribute to the resistant phenotype. While changes in TGFβRI and ligand expression were noted earlier, core TGF‐β signaling components, including modulators, nuclear or cytoplasmic retention factors, and interaction partners [44, 45], did not exhibit significant alterations (Fig. S11G). A detailed analysis of cluster 2, which identified genes with selectively increased expression in Huh7‐TR cells (Fig. 5D), revealed that PLA2G4A, MARK1, and GRM8 were of particular interest, ranking among the top differentially expressed genes (Fig. 6A–C and Table S4). Consistent with the pattern in cluster 2, their expression was not significantly altered in TGF‐β‐treated Huh7 cells (Fig. S11H). Phospholipase A2 group IVA (PLA2G4A), or cPLA2α, is known to modulate TGF‐β‐induced EMT in epithelial HCC cells by activating the PI3K/Akt pathway and inhibiting the TGF‐β/Smad signaling [46]. Microtubule affinity regulating kinase 1 (MARK1) belongs to the AMP‐activated protein kinase (AMPK) family, whose members control protein metabolism, polarity, and energy homeostasis [47]. Previous reports highlighted that several AMPK‐related kinases can interact with Smad proteins and regulate responses to TGF‐β signaling [48]. Functional validation through CRISPR/Cas9‐mediated depletion of PLA2G4A in Huh7‐TR cells resulted in a modest yet significant resensitization in TGF‐β cytostatic responses, as evidenced by a reduction in BrdU incorporation (Fig. 6D,E), suggesting that PLA2G4A contributes to the attenuation of TGF‐β's growth‐inhibitory signals in these cells. To elucidate the functional implications of MARK1 in modulating TGF‐β responses, we generated MARK1 knockout Huh7‐TR cells (Fig. 6F). Upon subsequent TGF‐β stimulation, MARK1‐deficient cells demonstrated a marked reduction in DNA synthesis and proliferation rates (Fig. 6G). Concurrently, MARK1 depletion led to a significant enhancement of SA‐β‐gal activity upon TGF‐β treatment (Fig. 6H). At the molecular level, these phenotypic changes were associated with significant restoration of TGF‐β/Smad3‐mediated transcriptional responses, as evidenced by elevated activity of a Smad3‐responsive reporter construct (Fig. 6I). Most critically, a time‐resolved nucleocytoplasmic shuttling assay revealed that MARK1 depletion reinstated the nuclear translocation and retention dynamics of Smad2 and Smad3 upon acute TGF‐β stimulation (Fig. 6J,K and Fig. S12A,B). This reestablished Smad nuclear activity likely enables proper transcriptional reactivation, reinstating the cytostatic effects of TGF‐β signaling.

**Fig. 7:**
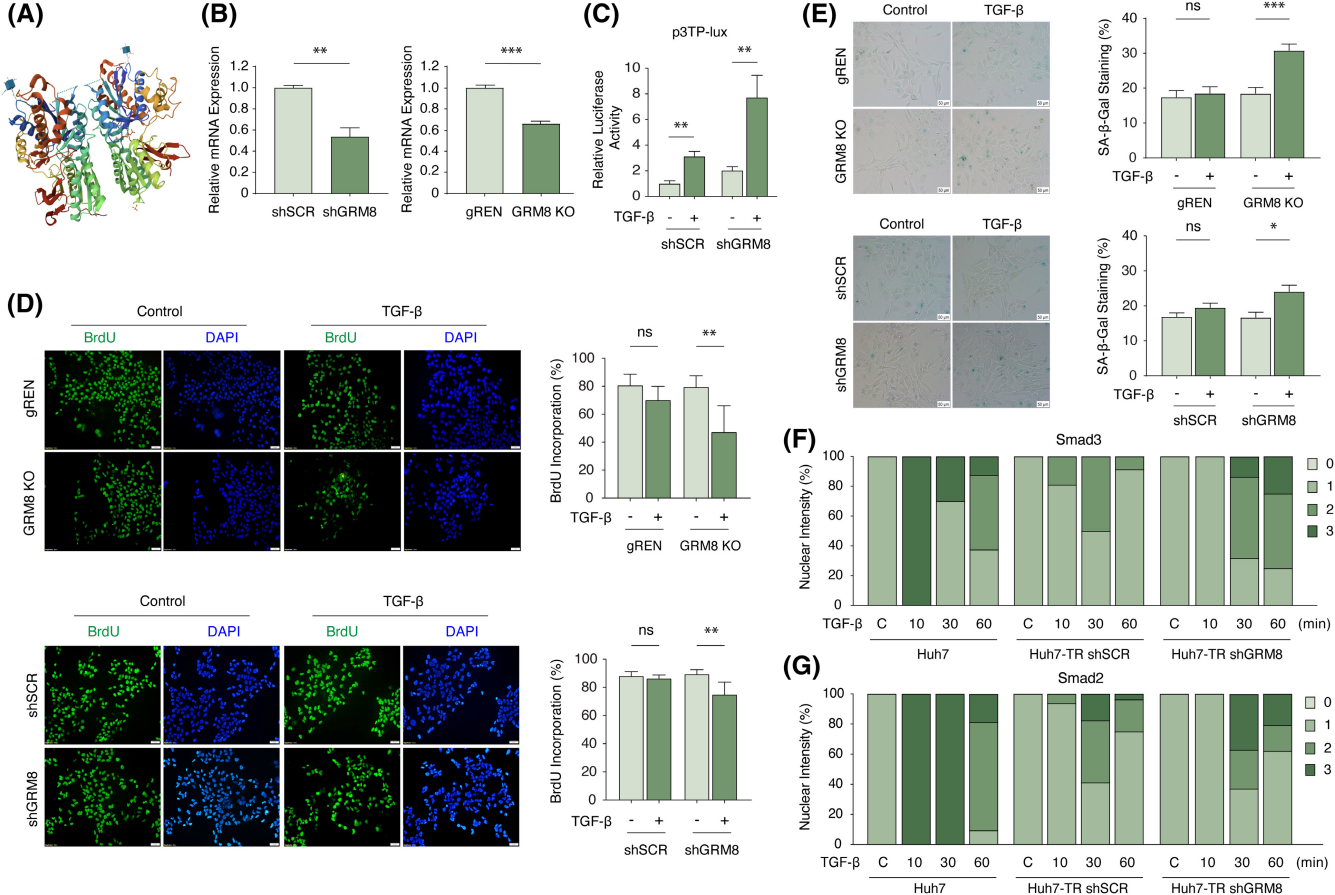
GRM8 impairs TGF‐β/Smad3 signaling activity in Huh7‐TR cells. (A) Druggable structure of GRM8, retrieved from CanSar. (B) Validation of GRM8 transcript levels through targeted manipulation using RNAi and CRISPR/Cas9 techniques in Huh7‐TR cells. (C) GRM8 attenuates TGF‐β/Smad3 transcriptional activity. Cells stably transduced either with pLKO.1 puro shSCR or pLKO.1 puro shGRM8 were co‐transfected with p3TP‐Lux and pRL‐TK plasmids and then left untreated or treated with TGF‐β (5 ng·mL^−1^) for 24 h. The luciferase activity was measured and expressed as fold change of Reporter/pRL‐TK. (D) BrdU incorporation assay identified a reduced cell proliferation index (BrdU positivity, 24‐h labeling) in GRM8 knockout (5 days TGF‐β) or knockdown (3 days TGF‐β) cells compared to control cells (gREN or shSCR, respectively). Percent BrdU was calculated by manual counting of 6 areas from each triplicate experiment. Scale bar: 50 μm. (E) SA‐β‐Gal activity identified increased cytostatic responses to TGF‐β activity in GRM8 knockout or knockdown cells compared to control cells (gREN or shSCR, respectively). Representative images are shown (SA‐β‐Gal staining in blue); scale bar: 50 μm. (F, G) GRM8 dysregulates time‐resolved signaling dynamics of nuclear Smad3 and Smad2. Huh7 cells were used as a control. All data in bar graphs are plotted as mean ± SD on three replicates. Statistical analyses were performed with Student's *t*‐test. *P*‐values, *P < 0.05, **P < 0.01, and ***P < 0.001; ns, not significant. BrdU, bromodeoxyuridine; CanSar, the cancer drug discovery platform; GRM8, glutamate metabotropic receptor 8; Huh7‐TR, TGF‐β resistant; SA‐β‐Gal, senescence‐associated β‐galactosidase; shSCR, short hairpin scrambled; TGF‐β, transforming growth factor‐β.

Metabotropic glutamate receptor 8 (GRM8), also known as mGluR8, belongs to the G‐protein coupled receptor 3 family and is primarily recognized for its role in modulating neurotransmission in the central nervous system [49]. However, studies identified activating mutations in GRM8 [50], implicating its involvement in the pathogenesis and progression of several malignancies, including neuroblastoma, lung cancer, glioma, and breast cancer [51, 52]. In HCC, higher GRM8 expression was associated with poor prognosis [53]. On a molecular level, GRM8 activation is coupled to second messenger mechanisms, thereby modulating cAMP/PKA or MAPK pathways [52]. GPCRs are considered viable therapeutic targets in cancer treatment, and GRM8 is no exception [54]. Analysis from the CanSAR database [55] highlights that GRM8 is a druggable molecule, featuring a ligandable structure and known bioactive compounds (Fig. 7A). Through targeted manipulation using RNAi and CRISPR/Cas9 techniques (Fig. 7B and Fig. S13A,B), we demonstrated that increased GRM8 expression impedes TGF‐β/Smad3 transcriptional activity (Fig. 7C) and mitigates cytostatic responses to TGF‐β exposure (Fig. 7D,E). Mechanistically, similar to MARK1, depletion of GRM8 was able to restore nuclear signaling dynamics of Smad molecules, which potentially explains transcriptional reactivation and antiproliferative responses. Specifically, we observed that Smad3 was retained longer in the nucleus following TGF‐β stimulation (Fig. 7F and Fig. S13C). Similarly, Smad2 exhibited restored nuclear localization kinetics in GRM8‐depleted cells, albeit to a lesser extent (Fig. 7G and Fig. S13D). These findings suggest that GRM8 contributes to resistance mechanisms in TGF‐β signaling by modulating Smad dynamics. Consequently, targeting GRM8 and similar regulatory genes may represent a viable strategy for reactivating sensitivity to the tumor‐suppressive and pro‐senescent effects of the TGF‐β/Smad3 axis.

## 4 Discussion

TGF‐β signaling and cellular senescence are key hallmarks of HCC development and progression [56, 57]. In epithelial HCC cells, TGF‐β‐induced senescence serves as an anti‐tumor mechanism. Paradoxically, chronic elevation of TGF‐β is positively correlated with EMT, aggressiveness, and poor prognosis in advanced tumors. The mechanisms governing the profound transition of TGF‐β signaling outcomes, between growth suppressive and aggressive states, are ill‐defined. Most critically, whether this transition involves modulating the senescence response remains elusive. Building upon our current and previous findings showing that TGF‐β is a strong inducer of senescence in epithelial HCC cell lines [12], but not in mesenchymal HCC cell lines [17], we decided to expose the epithelial Huh7 and Hep3B cell lines to chronic TGF‐β treatment. Interestingly, prolonged TGF‐β exposure has been shown recently to induce early and deep senescence states in multiple cancer types. Notably, upon withdrawal of TGF‐β, SA‐β‐Gal–high senescent cells are able to resume proliferation in both states, albeit with deep senescent cells exhibiting a reduced capacity for reentry into proliferation [7].

In Huh7 cells, continuous TGF‐β exposure results in the evasion of the cellular senescence phenotype, concurring with clonal regrowth, persistent and nearly complete loss of TGF‐β transcriptional activity and tumor‐suppressive functions, and a significant decrease in epithelial cell traits. A similar phenomenon was observed in the Hep3B cell line, providing generalizability to our findings and suggesting a broader relevance across epithelial HCC models. The resistance to TGF‐β–induced senescence in Huh7‐TR cells appears specific, as doxorubicin treatment continues to elicit robust growth arrest and elevated SA‐β‐Gal activity, thereby negating a generalized impairment of senescence pathways. A critical consideration remains whether the observed phenotype emanates from a pre‐existing subpopulation within parental cells. Although the presence of inherently TGF‐β–tolerant cells cannot be entirely excluded, our data suggest that this is unlikely to be the predominant mechanism. If such a resistant subpopulation existed, the earlier emergence of resistant colonies would be expected. However, clonal regrowth manifests only after approximately 3 weeks of continuous exposure, aligning with reports that underscore cellular adaptation to chronic stressors through extensive genomic, transcriptomic, and metabolomic reprogramming over prolonged periods [1]. Furthermore, if pre‐existing resistant cells are present, they are likely to be rare and may be detectable through lineage tracing using genetic barcoding [58]. Therefore, we postulate that resistance arises through TGF‐β–induced early senescence or via phenotypic adaptations that confer the capacity to persist beyond deep senescence.

The acquisition of mesenchymal features in TGF‐β resistant cells results in enhanced migration, invasion, and metastasis in zebrafish xenograft models. Several attributes of Huh7‐TR cells are reminiscent of mesenchymal HCC cell lines, including suppression of TGF‐β cytostatic responses and increased autocrine production of TGF‐β ligands [17, 59]. It is thus plausible to speculate that Huh7 cells transition from an epithelial to a mesenchymal state. The enrichment of EMT and angiogenesis gene signatures in Huh7‐TR cells reinforces the notion that TGF‐β resistance is associated with a more invasive and metastatic phenotype. Indeed, dedifferentiation is often linked to increased malignancy, therapeutic resistance, and poor clinical outcomes in HCC [7]. While the EMT‐like phenotype has been proposed as a mechanism to bypass failsafe programs [60], our findings suggest that EMT may not be a prerequisite for the onset of TGF‐β insensitivity but rather a corollary of adaptive plasticity in response to chronic TGF‐β exposure. Resistance is also associated with a partial increase in 2D clonogenic capacity while demonstrating decreased abilities in 3D anchorage‐independent growth and spheroid formation. Notably, despite the absence of stemness hallmarks which typically confer resistance against DNA‐damaging agents [61, 62] and TGF‐β [63], the mixed EMT phenotype in Huh7‐TR cells is coincident with lower clonogenicity and a reduced capacity for spheroid growth in the absence of attachment. These findings are consistent with the well‐known fact that mesenchymal HCC cells poorly form tumor xenografts in mice relative to their more epithelial counterparts [64].

In line with current understanding, the concomitant enrichment of senescence and EMT gene signatures in senescent cells indicates the potential coexistence of both phenotypes within the same cellular milieu [65]. This further implies that cells undergoing senescence may adaptively exploit the EMT program to acquire a more aggressive phenotype characterized by increased motility and invasiveness [60]. Additionally, the overlapping KRAS gene signature offers insights into the similarities between the transcriptomic programs of TGF‐β‐induced and oncogene‐induced senescence, suggesting the activation of shared molecular pathways in response to different stimuli. Our GSEA data further highlight the activation of inflammatory pathways, including IL6/JAK/STAT3 and TNF‐α/NF‐κB signaling in senescent cells. These pathways are integral components of the SASP, which reinforce senescence in an autocrine manner and can exert pro‐tumorigenic effects on neighboring cells [66]. Although these pathways are also enriched in Huh7‐TR cells, the resistant state exhibits a distinct secretome that differs from both parental and senescent cells. The enrichment of TGF‐β signaling in the senescent state aligns with the role of TGF‐β ligands as both inducers and effectors of senescence, primarily through the activation of SMAD‐dependent transcriptional responses that enforce growth arrest [67]. Moreover, altered metabolic traits in the senescent state may also contribute to the development of a pro‐inflammatory SASP or help senescent cells adapt to the chronic TGF‐β stress.

The loss of TGF‐β sensitivity correlates with dysregulated expression of multiple signaling components, including TGF‐β receptors and Smad3. Additionally, the spatiotemporal dynamics of Smad2/3 and Smad4 molecules are impaired in Huh7‐TR cells. This is characterized by delayed nuclear localization or accelerated nuclear exclusion, which aligns with studies demonstrating that the balance between nuclear import and export of Smad proteins is critical for the amplitude and duration of TGF‐β signaling responses. Disruption of this balance can lead to attenuated signaling and contribute to cellular resistance to TGF‐β [68]. The upregulation of signaling components by TGF‐β in parental cells accentuates the prominent role of the TGF‐β/Smad3 signaling axis in senescence induction. This is further supported by the restoration of TGF‐β sensitivity in resistant cells upon forced expression of Smad3 or TGFβRI. Arguably, such deregulations compromise the ability of the Smad3/4 complex to fully engage in target gene expression. Although Smad3 functions are modulated through phosphorylation of the linker region by intracellular kinases [69], and previous studies in HCC tumor models indicate that Smad3 linker phosphorylation attenuates TGF‐β transcriptional activities, thereby facilitating the transition between TGF‐β‐mediated growth suppression and oncogenic signaling [70], it appears that this mechanism contributes minimally to the resistance phenomenon. Furthermore, dephosphorylation of Smad3 and its dissociation from nuclear retention factors are essential for nuclear export [71]. However, our data suggest that phosphatases may not be the primary mediators of this process, as their expression levels are unchanged. In TGF‐β‐stimulated resistant cells, the majority of inactive Smad3 localizes to the cytoplasm, suggesting limited translocation into the nucleus. This indicates that inactive Smad3 may be sequestered in the cytoplasm, away from receptors, potentially through interactions with cytoplasmic retention factors. Importantly, Smad Anchor for Receptor Activation (SARA), a candidate cytoplasmic retention factor [72], appears unlikely to act as the endogenous retention factor for Smad3, given its stable expression levels. An alternative mechanism may involve the microtubule network, which has been shown to interact with Smads 2, 3, and 4, regulating their transcriptional activity and responsiveness to TGF‐β exposure [73]. Thus, an intact microtubule network characterized by inherent dynamic stability and responsiveness to both intrinsic and extrinsic cues may constitute a key regulatory component of the TGF‐β/Smad signaling axis.

Considering the substantial variations in transcriptome profiles between different states, we infer that TGF‐β resistance involves the simultaneous activation of multiple genes or pathways. Our studies implicate the TGF‐β‐related molecule PLA2G4A in this process [46], and additionally probe MARK1 and GRM8 as novel genes capable of modulating the TGF‐β/Smad3 signaling outputs. MARK1, a member of the AMPK family, is involved in regulating cell polarity, microtubule dynamics, and energy homeostasis [74]. Given that Smad proteins are substrates of AMPK‐related kinases [75], it is plausible that MARK1 interacts with Smad3 or modulates microtubule functions, thus affecting the trafficking of Smad molecules. Similarly, GRM8, a metabotropic glutamate receptor, is a G‐protein coupled receptor implicated in various cancers [76]. GRM8 activation is known to influence intracellular signaling cascades, leading to decreased cyclic AMP (cAMP) levels and modulation of downstream effectors such as protein kinase A (PKA) [52]. PKA is recognized for its role in altering microtubule dynamics in cancer cells [77]. A functional interaction between TGF‐β and PKA signaling is critical for the execution of TGFβ‐mediated cellular responses [78]. Additionally, GRM8 has been shown to activate MEK/ERK signaling pathways [52], which engage in cross‐talk with components of TGF‐β signaling. Further investigation is obviously necessary to confirm the identity of the putative factors that modulate nucleocytoplasmic shuttling dynamics of Smad3. Mechanistic studies, supported by longitudinal epiregulome and gene expression profiling, along with high‐throughput assays, such as proteomics, drug or CRISPR screens, may help inform a better understanding of how these and potentially other genes contribute to escaping senescence and committing to resistance.

## 5 Conclusions

The work presented here provides evidence that chronic TGF‐β exposure promotes senescence plasticity, representing a critical phenomenon that may confer epithelial HCC cells an advantage to evolve towards a more aggressive phenotype. This is tightly associated with the loss of TGF‐β sensitivity, coinciding with an EMT‐like transformation, increased invasion, and acquisition of metastatic capabilities. Our findings help further explain the differential responses of epithelial and mesenchymal HCC cell lines to TGF‐β treatment. Additionally, our work has implications for the potential restoration of the TGF‐β/senescence axis in advanced HCCs with elevated TGF‐β, which offers a promising avenue for exploration, particularly in the context of anti‐tumor mechanisms and senolytic or senostatic therapies.

## Conflict of interest

The authors declare no conflict of interest.

## Author contributions

MK and DD conducted experiments, analyzed data, and created figures. SE analyzed RNA‐seq data, generated relevant plots and figures, and contributed to drafting the manuscript. BD, EC, MB, and MU conducted experiments and performed data analysis. GC‐A supervised zebrafish xenograft studies. FL contributed to data analysis, interpretation of results, and provided critical reagents. MO and SS conceptualized the study and acquired funding. GK carried out the principal computational work for RNA‐seq data. SS developed the methodology, performed experiments, and supervised the study. MK and SS wrote the manuscript. All authors read and approved the final version of the manuscript.

## Supporting information


**Fig. S1.** Huh7 cell line is a robust model for TGF‐β‐induced cellular senescence.
**Fig. S2.** TGF‐β‐induced cellular senescence is mediated through Smad3, and continuous monitoring of senescent cells reveals clonal regrowth.
**Fig. S3.** Cell cycle distribution or senescence in Huh7 and Huh7‐TR cells under TGF‐β or Doxorubicin (Doxo) stimulation.
**Fig. S4.** Loss of TGF‐β responsiveness in Huh7‐TR cells.
**Fig. S5.** Chronic TGF‐β treatment in Hep3B cell line promotes TGF‐β resistance.
**Fig. S6.** Ectopic induction of EMT‐like phenotype fails to confer TGF‐β resistance in Huh7 cells.
**Fig. S7.** Effects of chronic TGF‐β exposure on stemness markers in senescent and resistant states.
**Fig. S8.** Signaling dynamics of TGF‐β/Smad3 axis in resistant cells.
**Fig. S9.** Loss of TGF‐β sensitivity is related to defective nuclear/cytoplasmic Smad4 signaling.
**Fig. S10.** Ectopic Smad3 or TGFβRI activity reinstates TGF‐β sensitivity in Huh7‐TR cells.
**Fig. S11.** RNA‐seq analysis reveals differential gene expression changes in senescent and resistant states compared to the TGF‐β sensitive state.
**Fig. S12.** MARK1 dysregulates signaling dynamics of Smad molecules.
**Fig. S13.** GRM8 attenuates TGF‐β/Smad signaling.

**Table S1.** Oligonucleotides for gRNA and shRNA cloning.
**Table S2.** Primer sequences for qRT‐PCR.
**Table S3.** Primary and secondary antibodies.
**Table S4.** Top 50 genes in Cluster 2, Huh7‐TR vs Huh7.

## Data Availability

RNA‐Seq data were deposited into the Gene Expression Omnibus (GEO) under accession number GSE261099 and are available at the following URL: https://www.ncbi.nlm.nih.gov/geo/query/acc.cgi?acc=GSE261099.
