# Supplementary material for: Escape from TGF‐β‐induced senescence promotes aggressive hallmarks in epithelial hepatocellular carcinoma cells
Source: Mol Oncol. 2025 Mar 14;19(9):2594–618. doi: 10.1002/1878-0261.70021 (PMC12420363; doi:10.1002/1878-0261.70021)
Supplement: Supplementary file 1 — Fig. S1. Huh7 cell line is a robust model for TGF‐β‐induced cellular senescence. Fig. S2. TGF‐β‐induced cellular senescence is mediated through Smad3, and continuous monitoring of senescent cells reveals clonal regrowth. Fig. S3. Cell cycle distribution or senescence in Huh7 and Huh7‐TR cells under TGF‐β or Doxorubicin (Doxo) stimulation. Fig. S4. Loss of TGF‐β responsiveness in Huh7‐TR cells. Fig. S5. Chronic TGF‐β treatment in Hep3B cell line promotes TGF‐β resistance. Fig. S6. Ectopic induction of EMT‐like phenotype fails to confer TGF‐β resistance in Huh7 cells. Fig. S7. Effects of chronic TGF‐β exposure on stemness markers in senescent and resistant states. Fig. S8. Signaling dynamics of TGF‐β/Smad3 axis in resistant cells. Fig. S9. Loss of TGF‐β sensitivity is related to defective nuclear/cytoplasmic Smad4 signaling. Fig. S10. Ectopic Smad3 or TGFβRI activity reinstates TGF‐β sensitivity in Huh7‐TR cells. Fig. S11. RNA‐seq analysis reveals differential gene expression changes in senescent and resistant states compared to the TGF‐β sensitive state. Fig. S12. MARK1 dysregulates signaling dynamics of Smad molecules. Fig. S13. GRM8 attenuates TGF‐β/Smad signaling. [file MOL2-19-2594-s002.docx]

**Escape from TGF-β-induced Senescence Promotes Aggressive Hallmarks in Epithelial Hepatocellular Carcinoma Cells**

Minenur Kalyoncu ^1^, Dilara Demirci ^1^, Sude Eris ^1,2^, Bengisu Dayanc ^1,2^, Ece Cakiroglu ^1,2^, Merve Basol ^1,2^, Merve Uysal ^1,2^, Gulcin Cakan-Akdogan ^1,3^, Fang Liu ^4^, Mehmet Ozturk ^5,6^, Gökhan Karakülah ^1,2^, Serif Senturk ^1,2,6,#^

^1^ Izmir Biomedicine and Genome Center, Izmir, Turkey

^2^ Department of Genomics and Molecular Biotechnology, Izmir International Biomedicine and Genome Institute, Dokuz Eylul University, Izmir, Turkey

^3^ Department of Biomedicine and Health Technologies, Izmir International Biomedicine and Genome Institute, Dokuz Eylul University, Izmir, Turkey

^4^ Center for Advanced Biotechnology and Medicine, Susan Lehman Cullman Laboratory for Cancer Research, Ernest Mario School of Pharmacy, Rutgers Cancer Institute of New Jersey, Rutgers, The State University of New Jersey, Piscataway, New Jersey

^5^ Department of Medical Biology, Izmir Tinaztepe University School of Medicine, Izmir, Turkey

^6^ Department of Molecular Biology and Genetics, Bilkent University, Ankara, Turkey

# Correspondence: Prof. Dr. Serif Senturk

Research Group Leader

Functional Cancer Genomics Group

Izmir Biomedicine and Genome Center

Dokuz Eylul University Health Campus

Mithatpasa St. No: 58/5

35340 Balcova, Izmir / Turkey

Phone 1: 90 (232) 412 65 14

Phone 2: 90 (232) 299 41 61

E-mail: [serif.senturk@ibg.edu.tr](mailto:serif.senturk@ibg.edu.tr)

<http://www.ibg.edu.tr>

<https://www.senturklab.com/>

**Supplementary Figures**

**
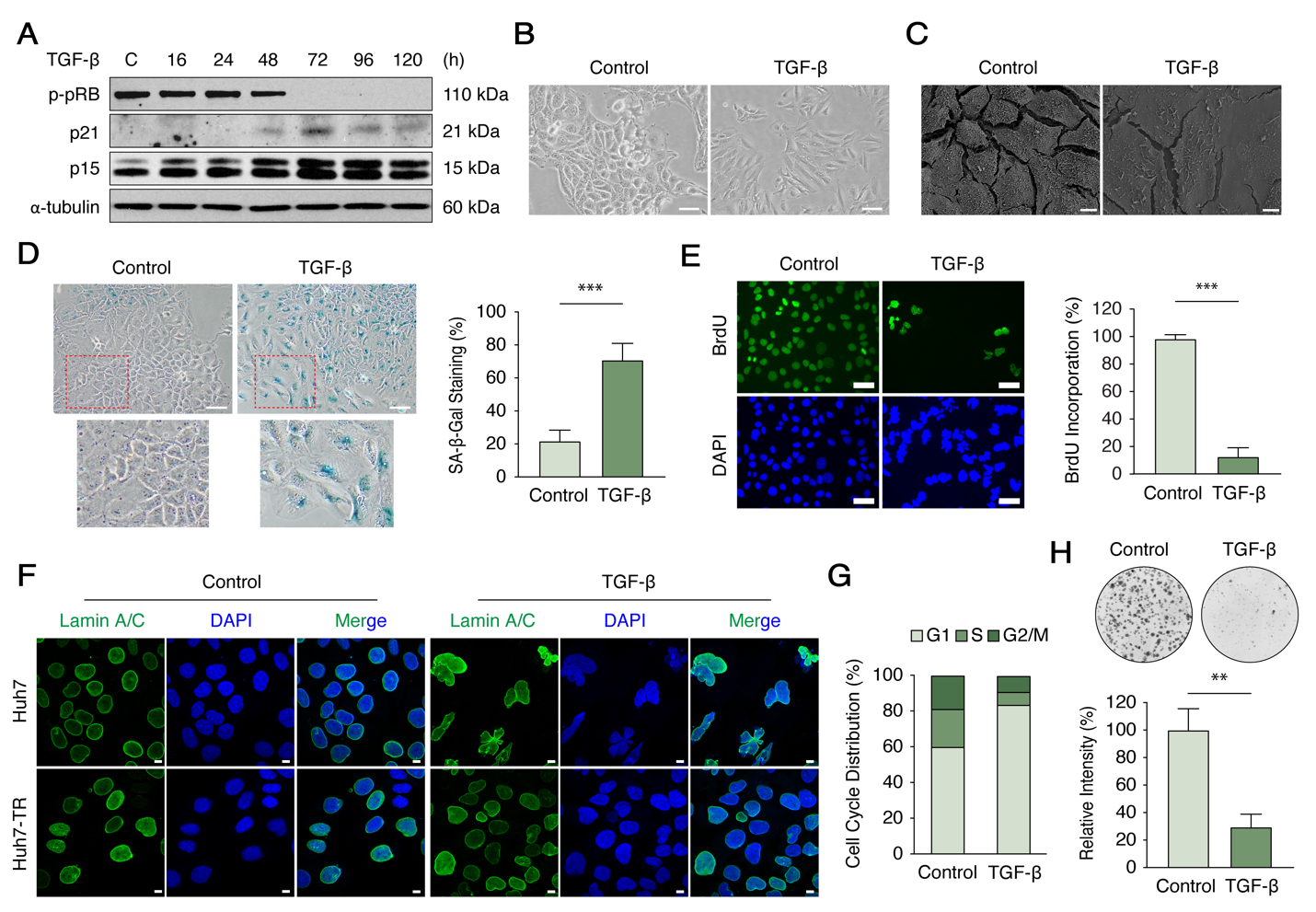
**

**Figure S1. Huh7 cell line is a robust model for TGF-β-induced cellular senescence. (A)** Time-resolved protein expression of known senescence markers. Huh7 cells were either left untreated (C: Control) or treated with 5 ng/mL TGF-β for indicated time points (h: hour). Western blotting analysis was performed with corresponding primary antibodies. α-tubulin served as the equal loading control. **(B, C)** Morphological changes in the Huh7 cell line after 72 h of TGF-β treatment. **(B)** Phase-contrast microscopy, scale bar: 100 µm. **(C)** Scanning electron microscopy (SEM), scale bar: 10 µm. **(D)** Induction of a strong cellular senescence response. Cells treated with TGF-β were assayed for SA-β-Gal activity. Representative images are shown (SA-β-Gal staining in blue), scale bar: 100 µm. Red rectangles represent focused inlets. (**E)** Immunofluorescence micrographs and quantification of BrdU staining (Green) on control and cells treated with 72 h of TGF-β. Nuclei were counterstained in blue with DAPI. Scale bar: 50 µm. (**F**) Senescent cells were exhibited by abnormal nuclear morphology. Representative images are shown Lamin A/C immunofluorescence staining. Scale bar: 10 µm. **(G)** 72 h of TGF-β treatment induces G1 arrest, accompanied by a strong attenuation in S phase. Cell cycle analysis was performed using PI staining. Bar graphs are presented as the mean of each cell cycle from three biological experiments. **(H)** 2D colony formation capacity of Huh7 cells following TGF-β treatment. Colony formation assay was performed in triplicates in 6-well cell culture plates for 14 days, followed by crystal violet staining. LI-COR Odyssey CLx Imaging System and Image Studio software were used to acquire high-resolution images of the plates and to measure signal intensities, respectively. Each of these panels **(D**, **E**, and **H)** are presented as the mean ± SD from three replicates. Statistical analyses were performed with two-tailed Student’s t-test. p values, TGF-β (5 ng/mL, 72 h) versus control; ** p < 0.01 and *** p < 0.001. Control: no TGF-β treatment. TGF-β, transforming growth factor-β; h, hour; SA-β-Gal, Senescence-associated β-galactosidase; BrdU, bromodeoxyuridine; PI, propidium iodide; 2D, two-dimensional.


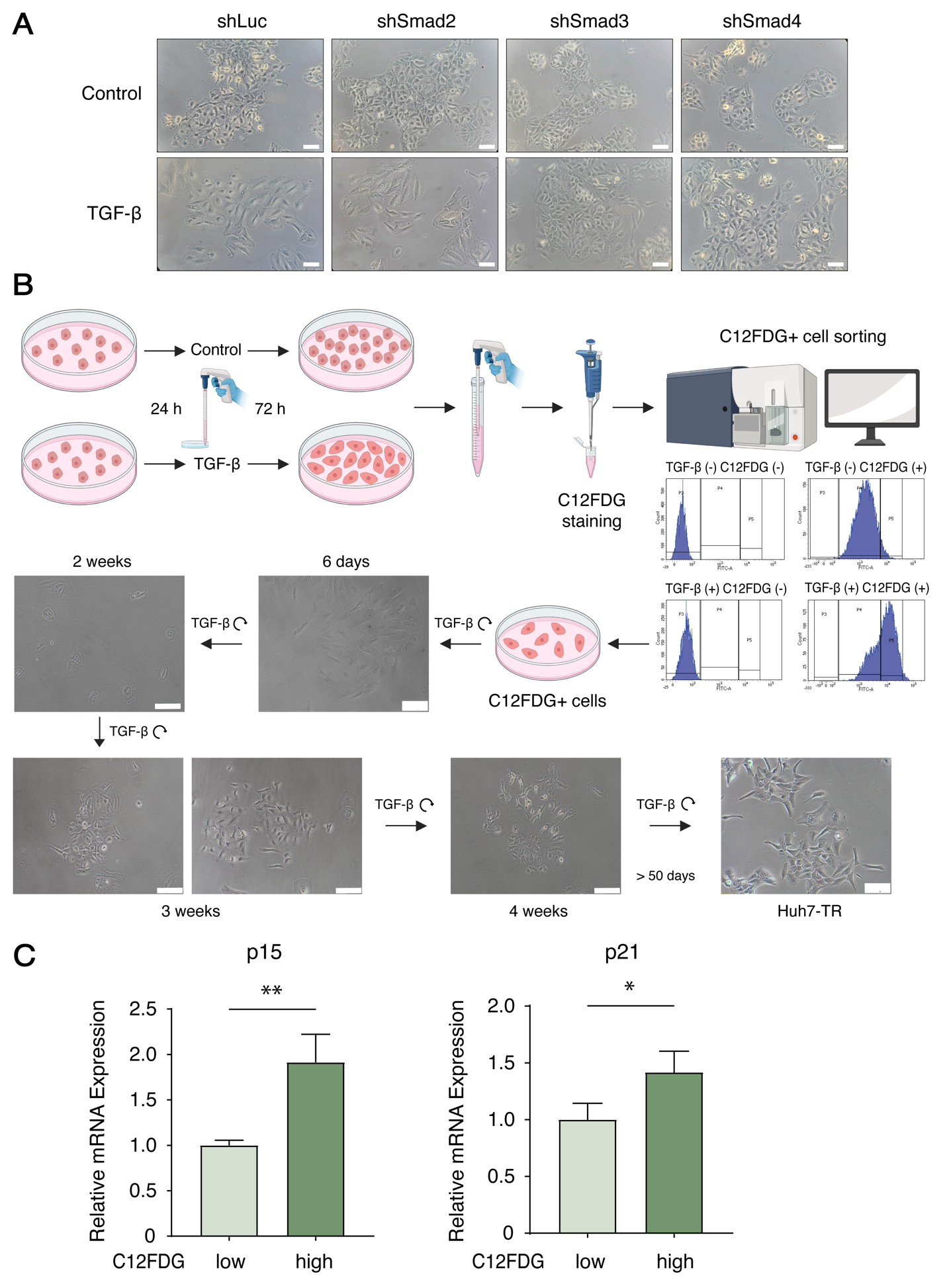


**Figure S2. TGF-β-induced cellular senescence is mediated through Smad3, and continuous monitoring of senescent cells reveals clonal regrowth. (A)** Phase-contrast microscopy images of Huh7 cells after 72 h TGF-β (5 ng/mL) treatment in Smad knockdown clones. Scale bar: 100 µm. **(B)** Schematic diagram of C12FDG staining, created with BioRender.com. Huh7 cells were treated with 5 ng/mL TGF-β for 72 h. Following treatment, cells were collected and stained with C12FDG for 1 h. C12FDG-positive (+) Huh7 cells were FACS-sorted and continued to receive chronic TGF-β (5 ng/mL). Phase-contrast microscopy, scale bar: 100 µm. **(C)** TGF-β-treated Huh7 cells (5 ng/mL, 3 days) were sorted after C12FDG staining. C12FDG-low and C12FDG-high populations, defined as the bottom 20% and top 30% of the observed distribution, respectively, were collected for gene expression analysis. Data in bar charts are presented as the mean ± SD from three replicates. Statistical analyses were performed with two-tailed Student’s t-test. p values, * p < 0.05 and ** p < 0.01. TGF-β, transforming growth factor-β; C12FDG, 5-dodecanoylaminofluorescein di-beta-D-galactopyranoside; h, hour; FACS, Fluorescence-activated cell sorting.


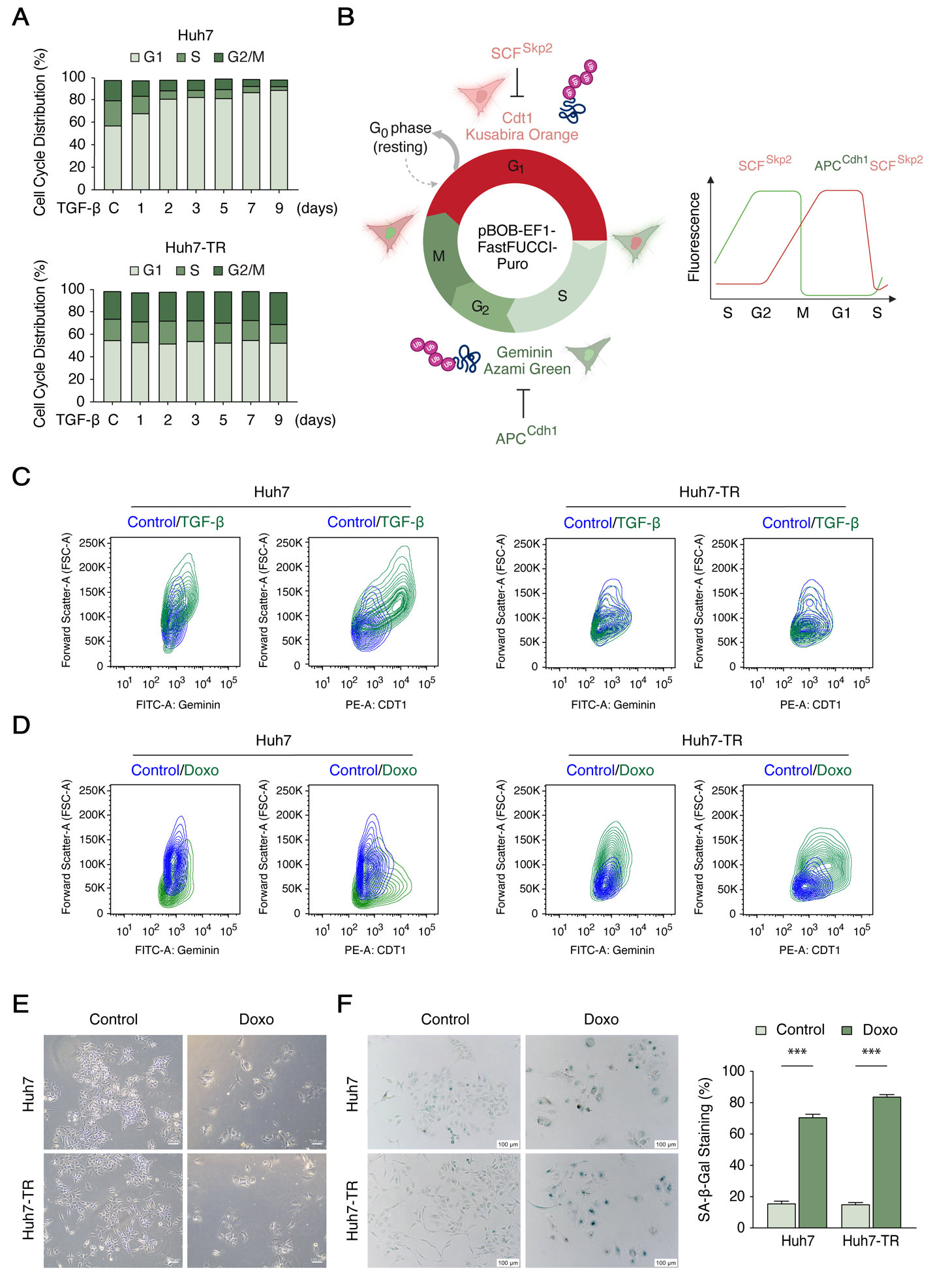


**Figure S3.** **Cell cycle distribution or senescence in Huh7 and Huh7-TR cells under TGF-β or Doxorubicin (Doxo) stimulation. (A)** Cell cycle analysis in Huh7 and Huh7-TR cells. Cells were either untreated (C: Control) or treated with TGF-β (5 ng/mL) for indicated time points (days). **(B)** Schematic diagram of FUCCI, created with BioRender.com. The FastFUCCI system is based on oscillating levels of SCF-Skp2 and APC/C (anaphase promoting complex/cyclosome) ubiquitin ligases, which target CDT1 and geminin, consecutively. In eukaryotic cells, CDT1 levels peak during G1 and plummet upon S entry. Conversely, the Geminin level is high during S and G2 but is low during late mitosis and G1. The degrons of CDT1 and Geminin are tagged with monomeric Kusabira orange (mKO2) and monomeric Azami green (mAG), respectively. Cells in G1 have a red fluorescence (Phycoerythrin (PE) and allophycocyanin (APC)), whereas remaining cells are tagged as green (and cells in transition can be observed as yellow), thus cells accumulated in different cell cycles can be monitored. **(C** and **D)** Cell cycle assessment was conducted on Huh7 and Huh7-TR cells stably engineered with the FastFUCCI system. An increase in CDT1 signal (represented in contour plots in the PE-A red channel, pseudocolored in green) following TGF-β (**C**) or Doxo (**D**) treatment indicates an accumulation in the G1 cell cycle phase. FITC-A green channel signals remain largely unaffected. **(E)** Phase-contrast microscopy images of Huh7 and Huh7-TR cells after 72 h Doxo (25 nM) treatment, scale bar: 100 µm. **(F)** Representative images of SA-β-Gal assay in Huh7 and Huh7-TR cells after 72 h of Doxo (25 nM) treatment. Scale bar: 100 µm. Data in bar charts are presented as the mean ± SD from three replicates. Statistical significance was calculated by a two-tailed Student’s t-test. p value, *** p < 0.001. Huh7-TR, TGF-β resistant; TGF-β, transforming growth factor-β; FUCCI, fluorescence ubiquitination-based cell cycle indicator; Doxo, Doxorubicin.


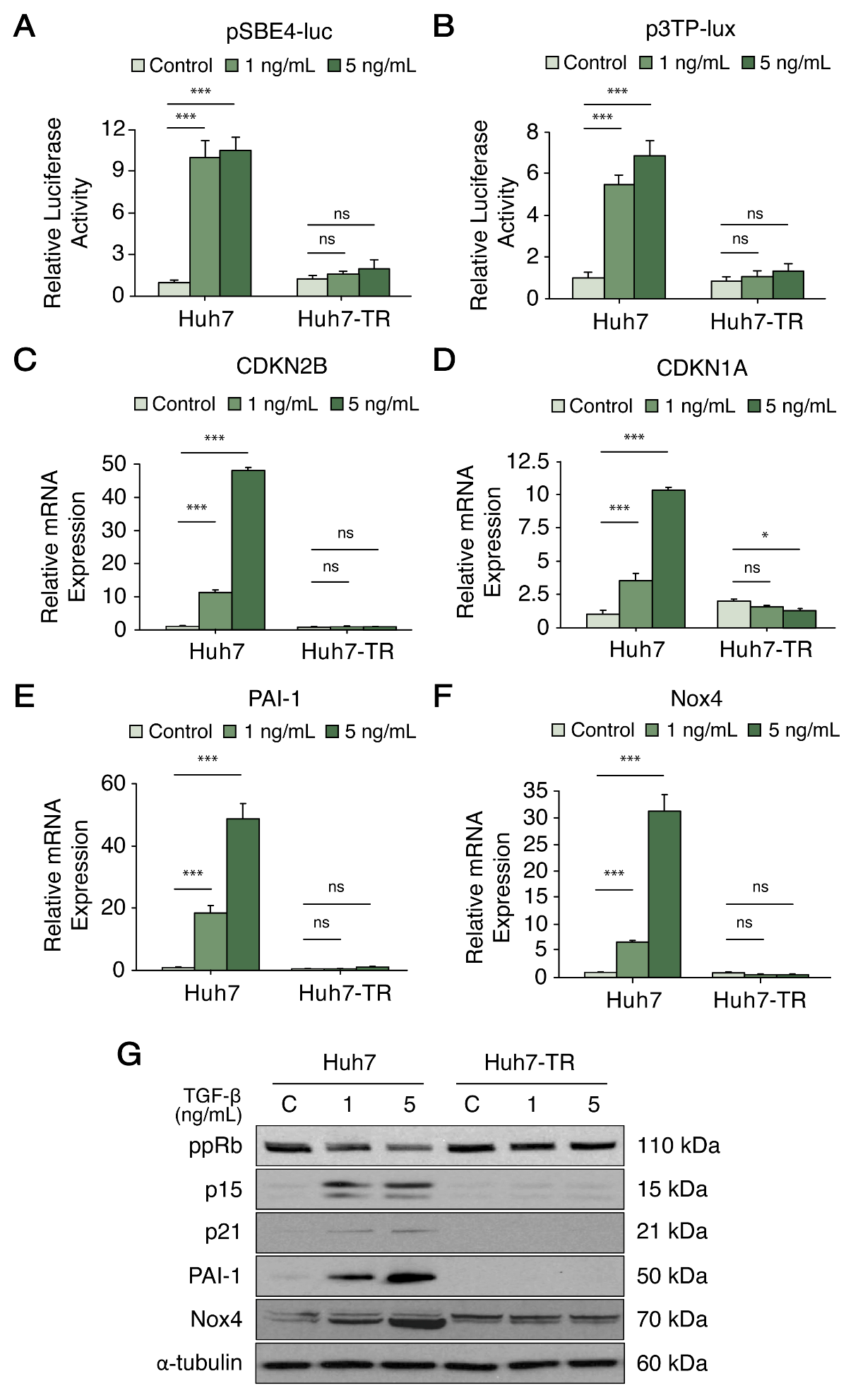


**Figure S4. Loss of TGF-β responsiveness in Huh7-TR cells. (A, B)** Diminished response to TGF-β treatment with pSBE4-luc and p3TP-lux reporters in Huh7-TR cells. Cells were co-transfected with pSBE4-Luc or p3TP-lux and control pRL-TK plasmids, and either left untreated or treated with TGF-β (1 ng/mL and 5 ng/mL) for 24 h. The luciferase activity was measured and expressed as fold change of Reporter/pRL-TK. Y-axis: Fold Change. **(C** to **F)** Expression of CDKN2B, CDKN1A, PAI-1 and Nox4 genes in Huh7-TR cells treated with 1 ng/mL and 5 ng/mL TGF-β for 72 h under normal culture conditions. qRT-PCR analysis was performed with gene-specific primer pairs. Expression values were normalized with respect to GAPDH. **(G)** Protein expression of target genes in parental Huh7 and Huh7-TR cells treated with 1 ng/mL and 5 ng/mL TGF-β for 72 h. Western blotting analysis was performed with corresponding primary antibodies. Same lysate samples from Fig. 3C were run in this western blot panel, hence the same α-tubulin equal loading was presented. Data in bar charts are presented as the mean ± SD from three replicates. Statistical significance was calculated by a two-tailed Student’s t-test. p values, * p < 0.05 and *** p < 0.001; ns: not significant. TGF-β, transforming growth factor-β; Huh7-TR, TGF-β resistant; h, hour; qRT-PCR, quantitative reverse transcription polymerase chain reaction.

**
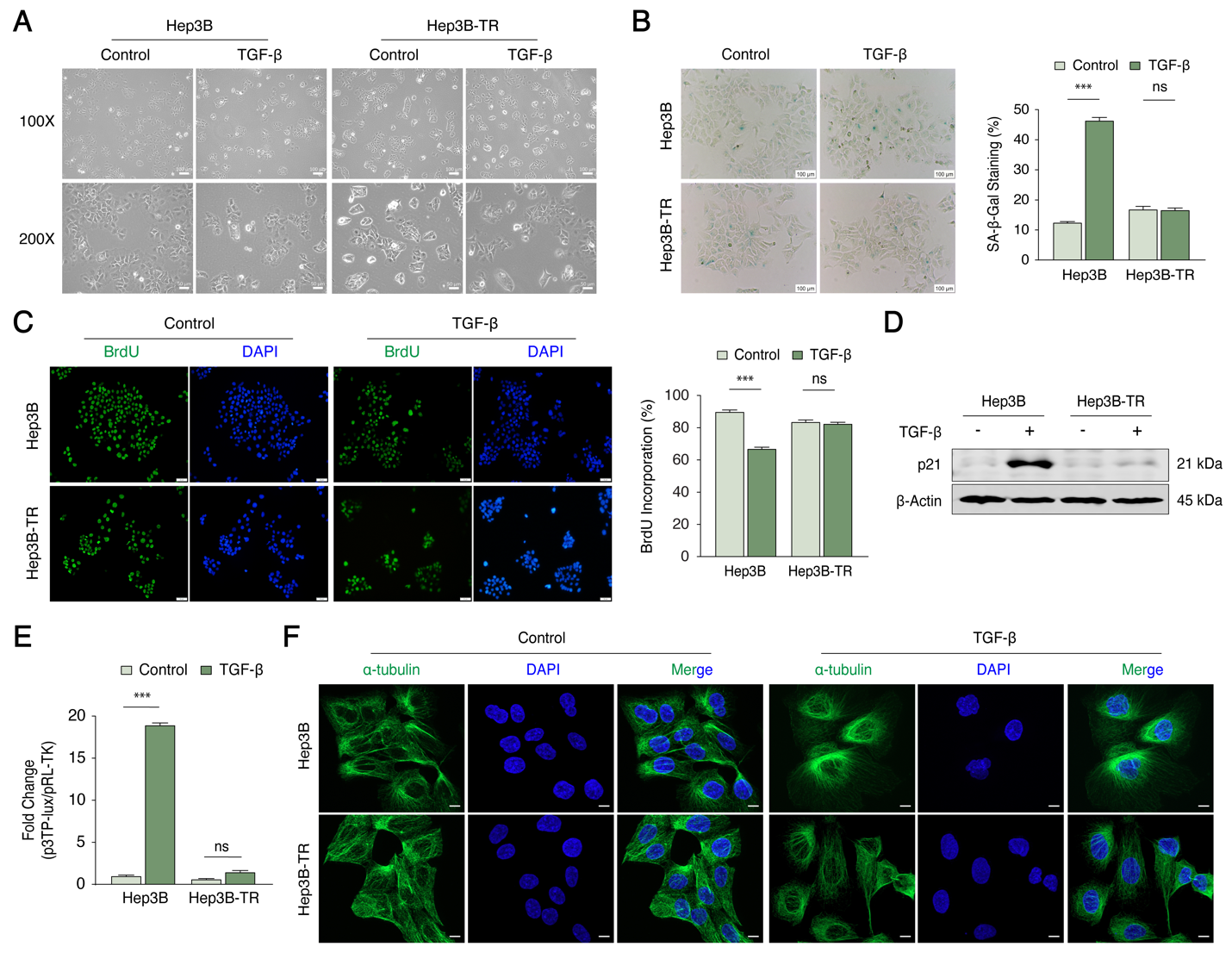
**

**Figure S5. Chronic TGF-β treatment in Hep3B cell line promotes TGF-β resistance. (A)** Morphological changes in Hep3B and Hep3B-TR cells after 72 h of TGF-β (5 ng/mL) treatment. Upper panel: 100X, scale bar: 100 µm; lower panel: 200X, scale bar: 50 µm. **(B)** Representative images of SA-β-Gal assay in Hep3B and Hep3B-TR cells after 72 h of TGF-β (5 ng/mL) treatment. Scale bar: 100 µm. **(C)** Resistance to inhibition of BrdU incorporation in Hep3B-TR cells. Cells were treated with 5 ng/mL TGF-β for 72 h. Cells positively labeled for BrdU (24 h) were stained with immunofluorescence technique using anti-BrdU (mouse) primary antibody which was followed by anti-mouse Alexa-488 secondary antibody incubation. Seven (7) areas were counted on each triplicate assay. Scale bar: 50 µm. (Control: no TGF-β treatment) **(D)** Protein expression of p21 in Hep3B and Hep3B-TR cells after 72 h treatment with TGF-β (5 ng/mL). Western blotting analysis was performed with corresponding primary antibodies. β-Actin served as the loading control. **(E)** Diminished response to TGF-β treatment with p3TP-lux reporter in Hep3B-TR cells. Cells were co-transfected with p3TP-lux and control pRL-TK plasmids, and either left untreated or treated with 5 ng/mL TGF-β for 24 h. The luciferase activity was measured and expressed as fold change of Reporter/pRL-TK. Y-axis: Fold Change. **(F)** Staining patterns of α-tubulin in Hep3B and Hep3B-TR cells. Scale bar: 10 µm. Data in bar charts are presented as the mean ± SD from three replicates. Statistical significance was calculated by a two-tailed Student’s t-test. p values, *** p < 0.001 and ns: not significant. TGF-β, transforming growth factor-β; h, hour; Hep3B-TR, TGF-β resistant; SA-β-Gal, Senescence-associated β-galactosidase; BrdU, bromodeoxyuridine.

**
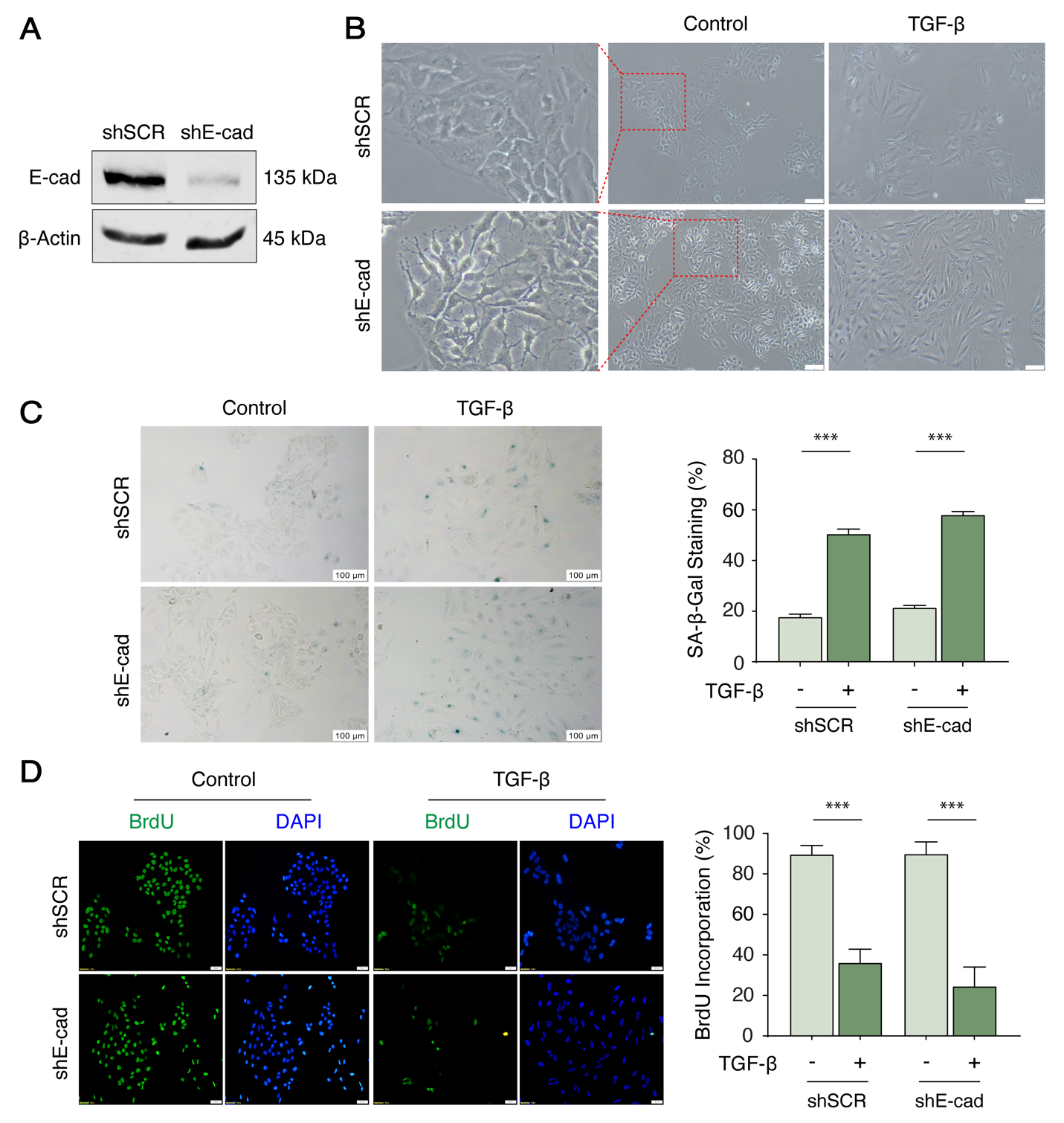
**

**Figure S6. Ectopic induction of EMT-like phenotype fails to confer TGF-β resistance in Huh7 cells. (A)** Western blot confirmation of E-Cadherin silencing via shRNA knockdown. β-Actin served as the loading control. **(B)** Morphological changes in Huh7 cells after E-Cadherin depletion and TGF-β treatment (5 ng/mL, 72 h). Scale bar:100 µm. Zoomed images are highlighted by red rectangles. **(C)** Representative images of SA-β-Gal assay in E-Cadherin knockdown cells compared to control cells after 72 h of TGF-β (5 ng/mL) treatment. Scale bar: 100 µm. Bar graphs are presented as the mean ± SD on three replicates. **(D)** BrdU incorporation assay identified reduced cell proliferation in E-Cadherin silenced cells. Percent BrdU was calculated by manual counting of 8 areas from each triplicate experiment. Scale bar: 50 µm. Statistical significance was performed by a two-tailed Student’s t-test. p value, *** p < 0.001. EMT, epithelial-to-mesenchymal transition; TGF-β, transforming growth factor-β; shRNA, short hairpin RNA; SA-β-Gal, Senescence-associated β-galactosidase; h, hour; BrdU, bromodeoxyuridine.

**
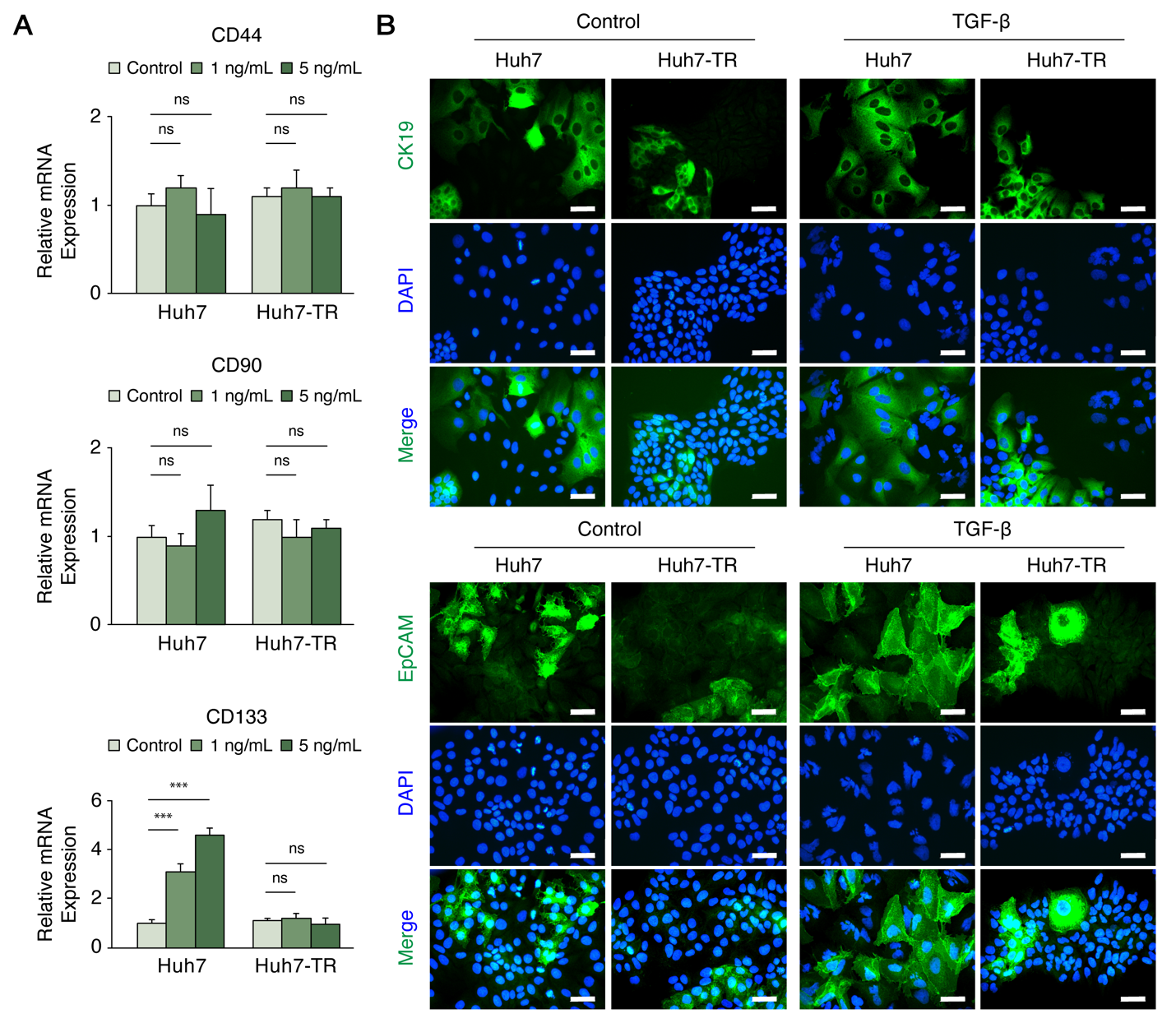
**

**Figure S7. Effects of chronic TGF-β exposure on stemness markers in senescent and resistant states. (A)** Expression of known putative cancer stem cell (CSC) markers, namely CD44, CD90, and CD133 in Huh7 and Huh7-TR. Cells were treated with 1 ng/mL and 5 ng/mL TGF-β for 72 h. All expression values were normalized with GAPDH. Control: no TGF-β treatment. Data in bar charts are presented as the mean ± SD from three replicates. Statistical significance was calculated by a two-tailed Student’s t-test. p values, *** p < 0.001 and ns: not significant. **(B)** Staining pattern of CK19 and EpCAM/ESA, defined as bipotential hepatic progenitor cell markers. Nuclei were counterstained in blue with DAPI. Scale bar: 50 µm. TGF-β, transforming growth factor-β; Huh7-TR, TGF-β resistant; h, hour.


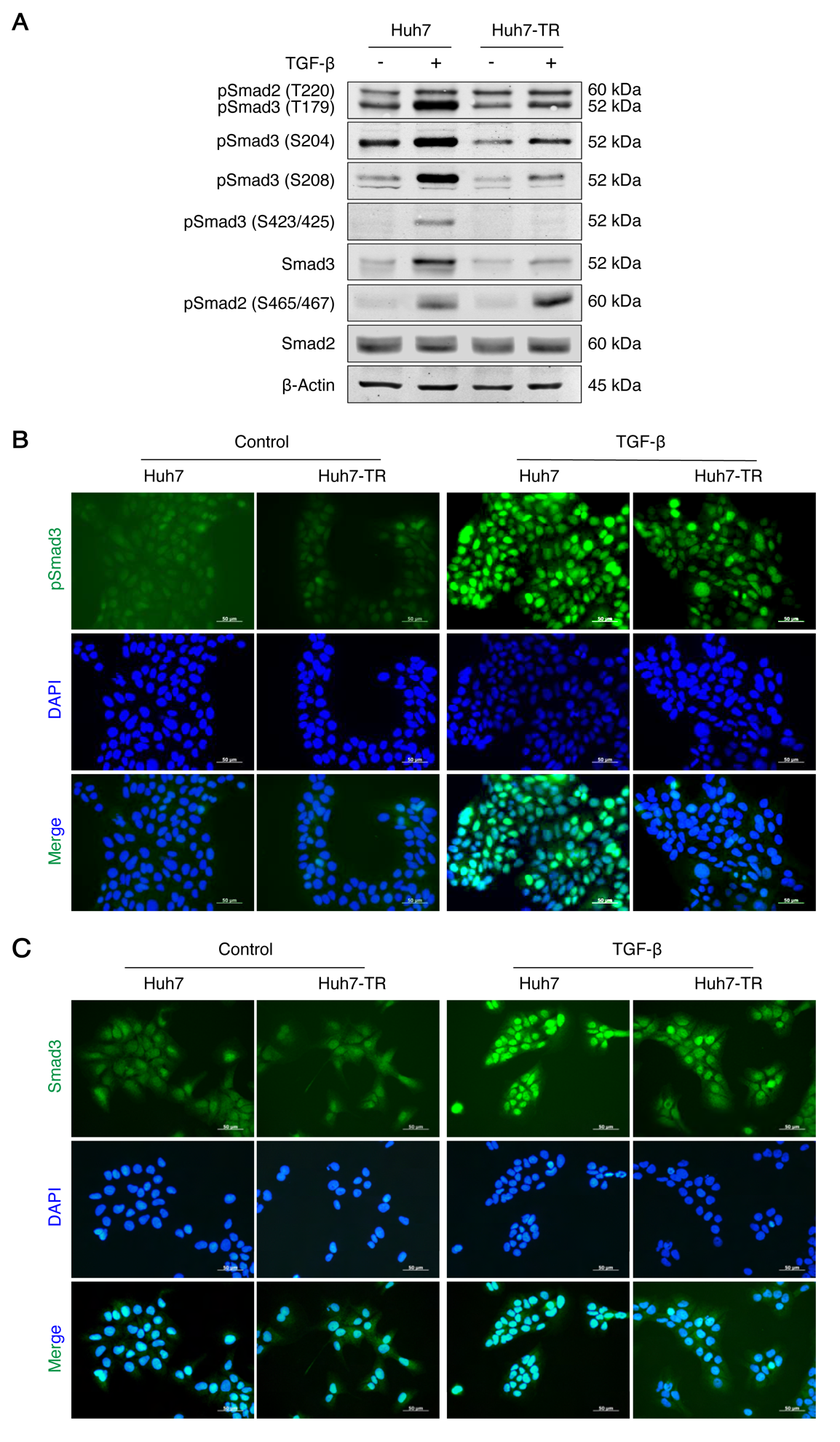


**Figure S8. Signaling dynamics of TGF-β/Smad3 axis in resistant cells. (A)** Protein expression of target molecules in Huh7 and Huh7-TR cells after 72 h treatment with TGF-β (5 ng/mL). Western blotting analysis was performed with corresponding primary antibodies. β-Actin served as the loading control. **(B, C)** Decreased nuclear accumulation of activated p-Smad3 **(B)** and total Smad3 **(C)** after 60 min of TGF-β treatment in Huh7-TR. Nuclei were counterstained in blue with DAPI. Scale bar: 50 μm. TGF-β, transforming growth factor-β; Huh7-TR, TGF-β resistant; h, hour; min, minute.

**
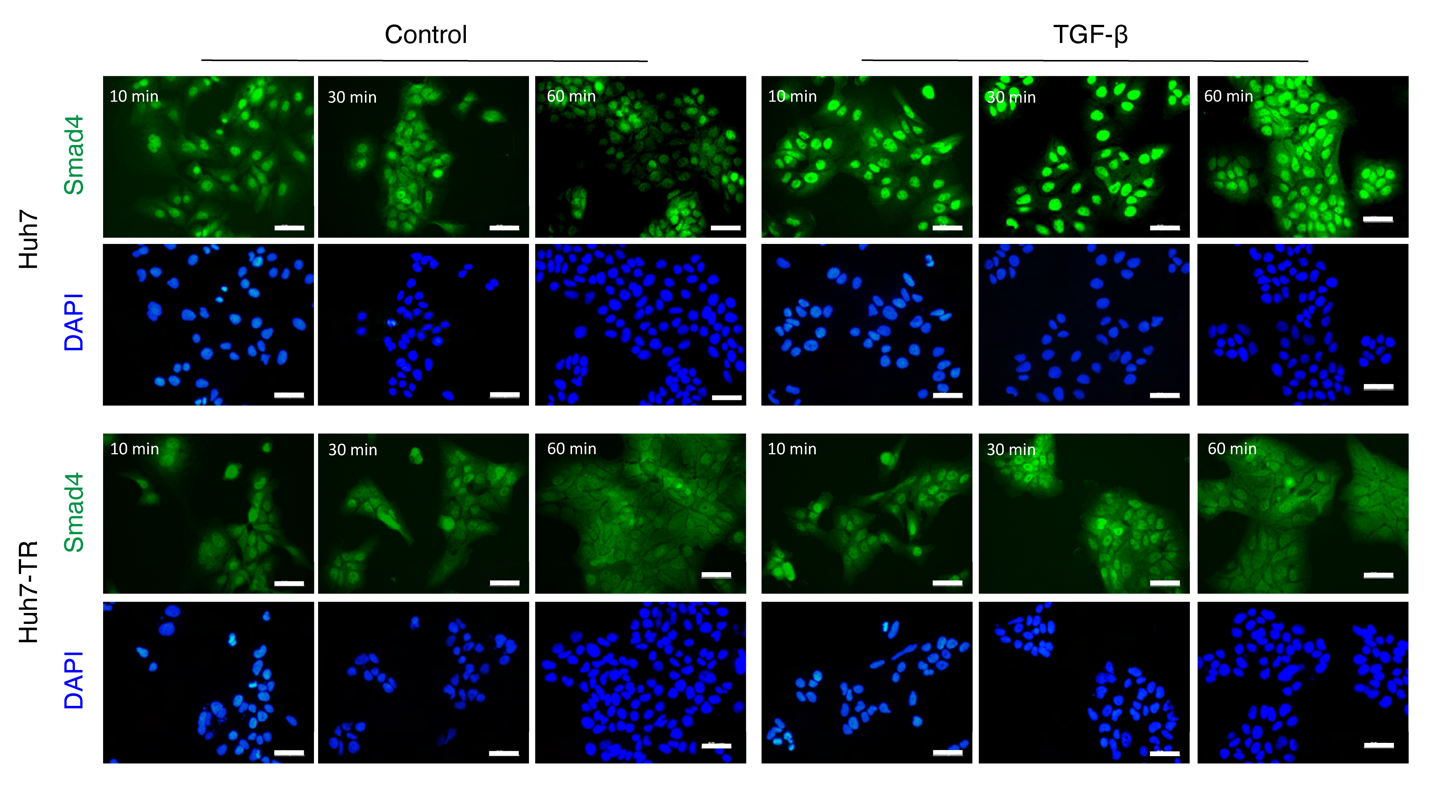
**

**Figure S9. Loss of TGF-β sensitivity is related to defective nuclear/cytoplasmic Smad4 signaling.** Diminished nuclear accumulation and time-dependent delay in Smad4 translocation in Huh7-TR. Cells were treated with TGF-β (5 ng/mL) for specified time periods. Nuclei were counterstained in blue with DAPI. Scale bar: 20 μm. TGF-β, transforming growth factor-β; Huh7-TR, TGF-β resistant.

**
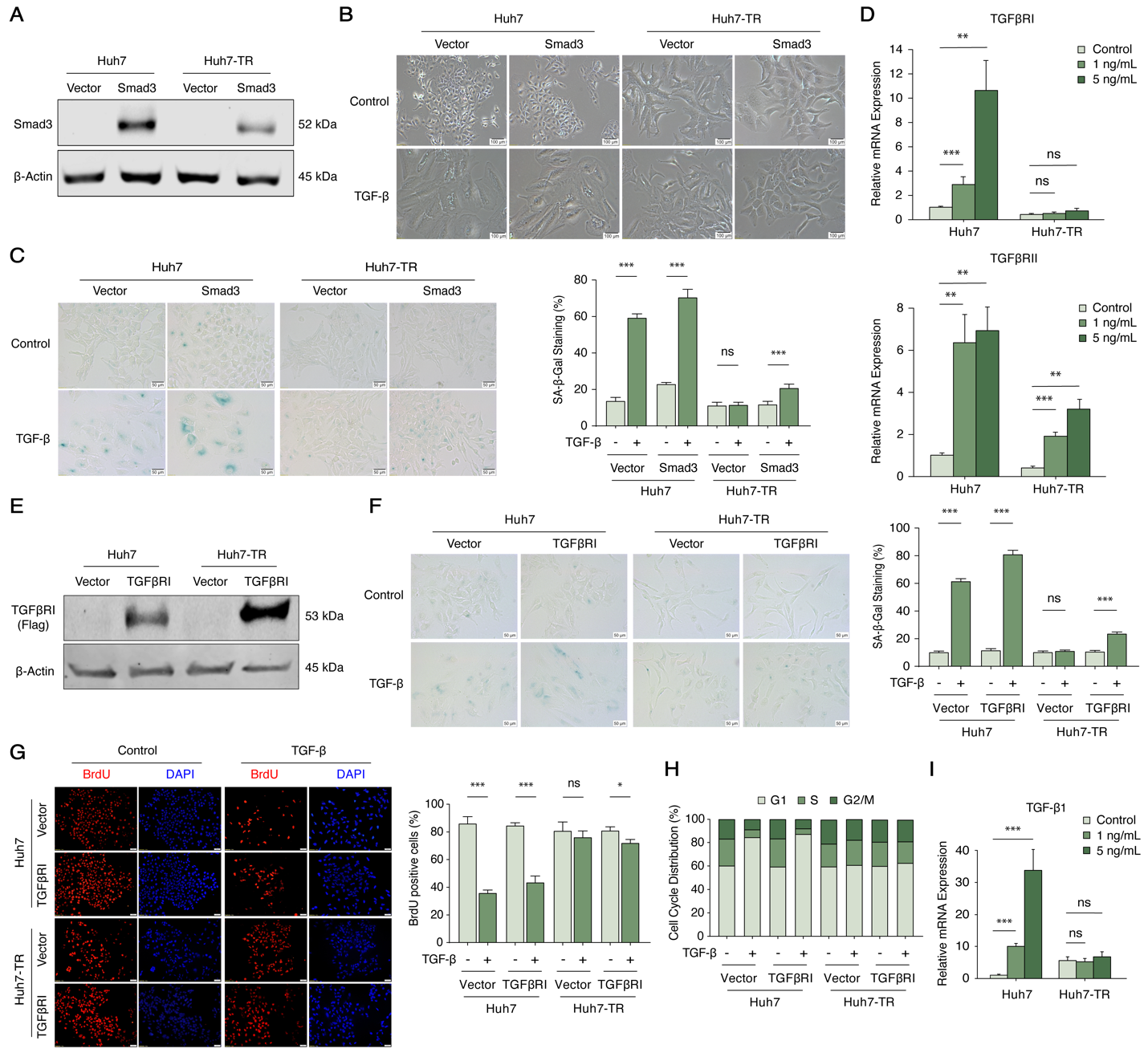
**

**Figure S10. Ectopic Smad3 or TGFβRI activity reinstates TGF-β sensitivity in Huh7-TR cells. (A)** Western blot confirmation of stable Smad3-overexpressing Huh7 and Huh7-TR cells. Western blotting analysis was performed with corresponding primary antibodies. β-Actin was monitored as equal loading. **(B)** Morphological changes in Smad3-overexpressing Huh7 and Huh7-TR cells after 5 days of TGF-β (5 ng/mL) treatment. Scale bar: 100 µm. **(C)** SA-β-Gal activity was notably increased in stable Smad3-overexpressing Huh7 and Huh7-TR cells after 5 days of TGF-β (5 ng/mL) treatment. Representative images are shown (SA-β-Gal staining in blue), scale bar: 50 µm. **(D)** Expression of TGF-β receptors in Huh7 and Huh7-TR. Cells were treated with 1 ng/mL and 5 ng/mL TGF-β for 72 h. qRT-PCR analysis was performed with gene-specific primer pairs. Expression values were normalized with GAPDH. **(E)** Western blot confirmation of stable TGFβRI-overexpressing Huh7 and Huh7-TR cells. Western blotting analysis of TGFβRI was performed using a Flag-tag specific antibody. β-Actin was monitored as equal loading. **(F)** SA-β-Gal activity was elevated in stable TGFβRI-overexpressing Huh7-TR cells after 72 h of TGF-β (5 ng/mL) treatment. Representative images are shown (SA-β-Gal staining in blue), scale bar: 50 µm. **(G)** BrdU incorporation assay identified reduced cell proliferation index in stable TGFβRI-overexpressing Huh7-TR cells. Representative images are shown (BrdU staining in red). Nuclei were counterstained in blue with DAPI. Scale bar: 50 µm. Percent BrdU was calculated by manual counting of 6 areas from each triplicate experiment. **(H)** Slight increase in G1 cell cycle arrest in stable TGFβRI-overexpressing Huh7-TR cells. Cell cycle analysis was performed using PI staining. Bar graphs are presented as the mean of each cell cycle from three experiments. **(I)** Marked upregulation of TGF-β1 ligand expression in Huh7-TR cells. Cells were treated with 1 ng/mL and 5 ng/mL TGF-β for 72 h. qRT-PCR analysis was performed with gene-specific primer pairs. Expression values were normalized with GAPDH. Data are plotted as mean ± SD on three replicates. Statistical analyses were performed with two-tailed Student’s t-test. p values, * p < 0.05 ** p < 0.01, *** p < 0.001 and ns: not significant. TGFβRI, transforming growth factor-β receptor I; TGF-β, transforming growth factor-β; Huh7-TR, TGF-β resistant; SA-β-Gal, Senescence-associated β-galactosidase; qRT-PCR, quantitative reverse transcription polymerase chain reaction; h, hour; BrdU, bromodeoxyuridine; PI, propidium iodide; TGF-β1, transforming growth factor-β1.

**
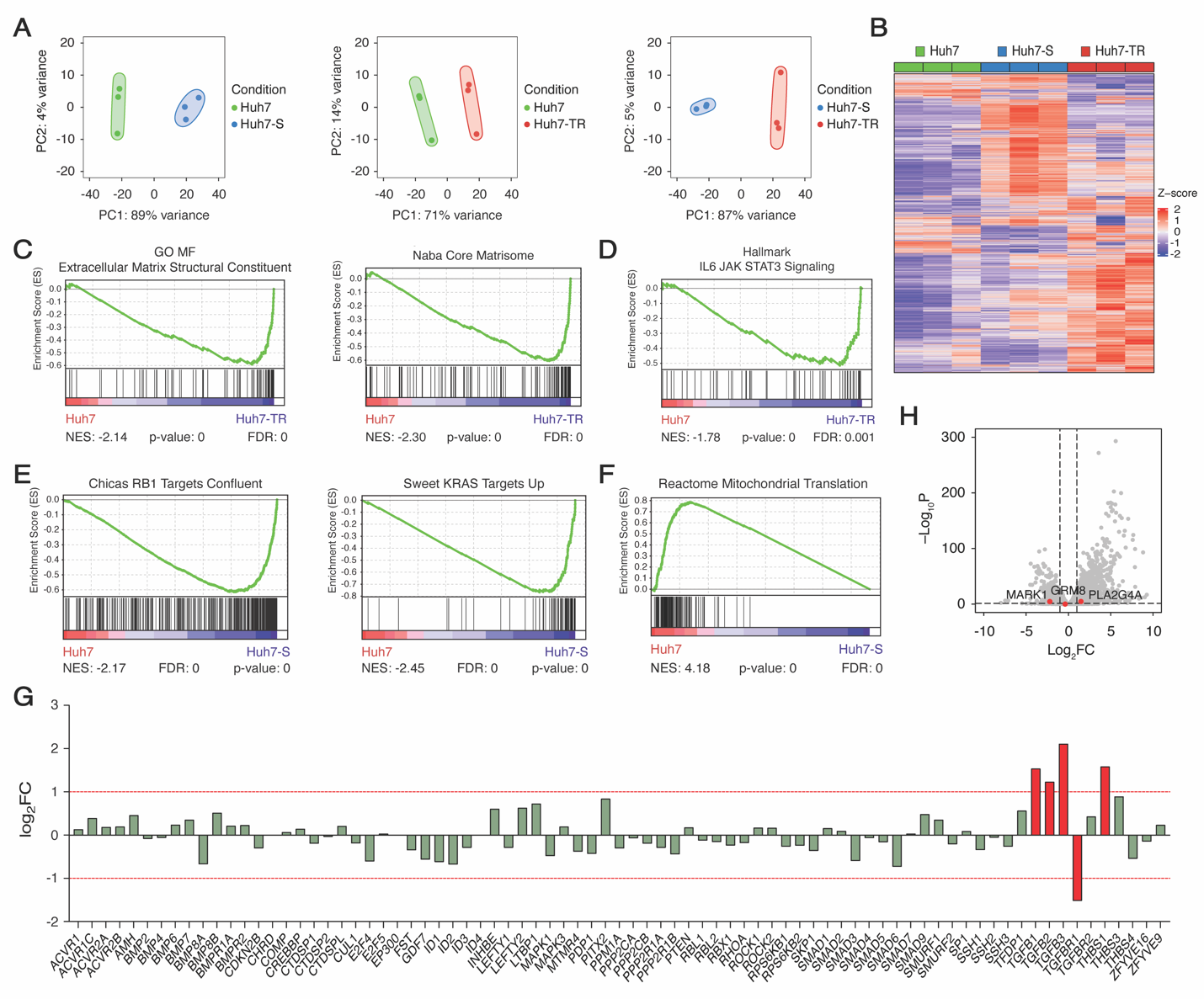
**

**Figure S11. RNA-seq analysis reveals differential gene expression changes in senescent and resistant states compared to the TGF-β sensitive state. (A)** Principal Component Analysis of RNA-Seq for Huh7 vs Huh7-S, Huh7 vs Huh7-TR, and Huh7-S vs Huh7-TR, respectively. **(B)** Whole genome transcriptome heatmap of gene expression across Huh7, Huh7-S, and Huh7-TR samples. **(C** to **F)** GSEA of select gene signatures based on differentially expressed genes between different states according to the RNA-seq data. ES represents enrichment score. **(G)** Bar plot showing log fold change values of TGF-beta signaling pathway genes in Huh7-TR vs Huh7 cells. Green bars represent genes within threshold limits (red dashed lines), while red bars indicate genes with significant expression changes above or below these thresholds. **(H)** Volcano plot representation of differentially expressed genes between Huh7-S vs Huh7 states. PLA2G4A, MARK1, and GRM8 genes are labeled with red dots. TGF-β, transforming growth factor-β; Huh7-S, TGF-β-induced senescence; Huh7-TR, TGF-β resistant; GSEA, gene set enrichment analysis; PLA2G4A, phospholipase A2 group IVA; MARK1, microtubule affinity regulating kinase 1; GRM8, glutamate metabotropic receptor 8.

**
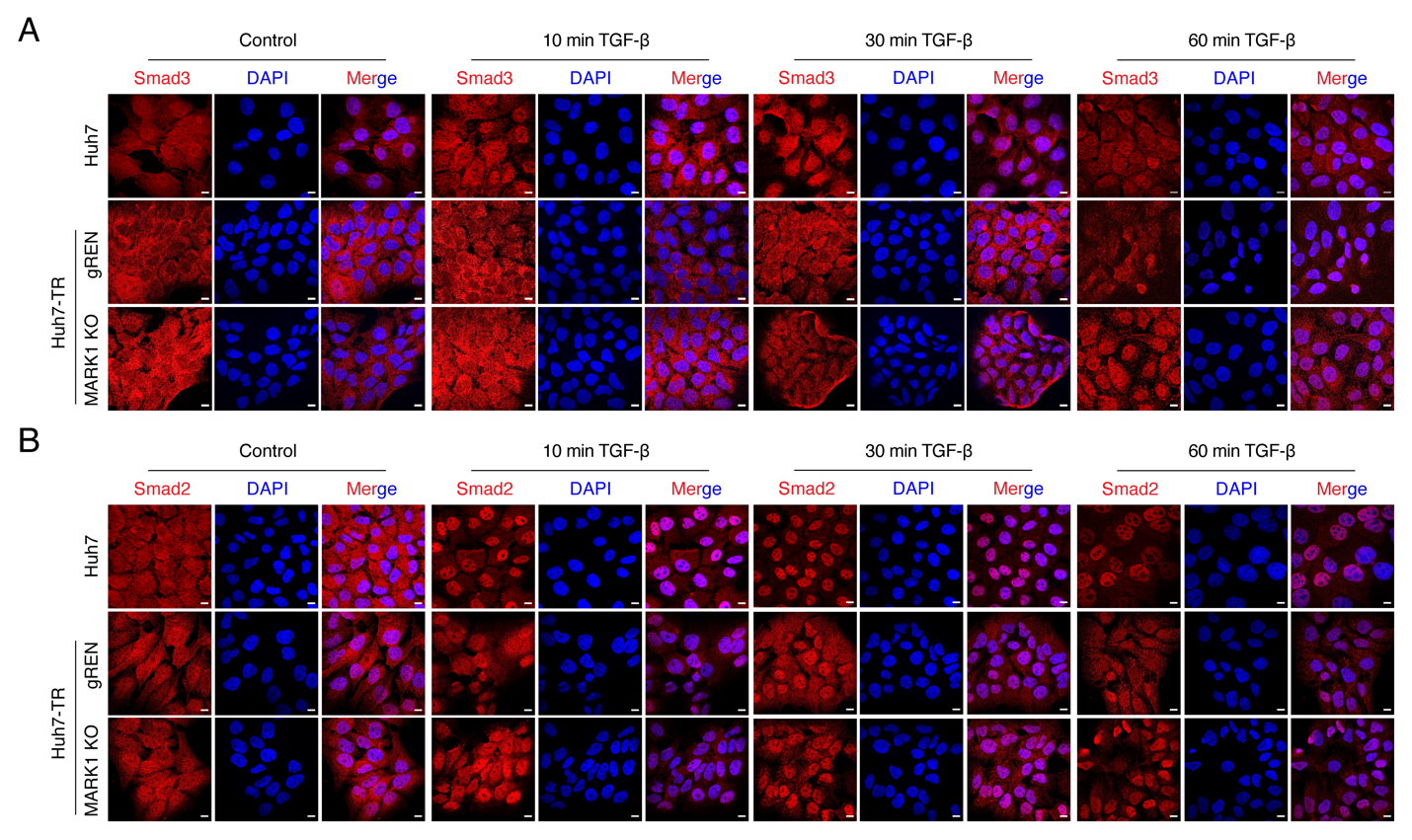
**

**Figure S12. MARK1 dysregulates signaling dynamics of Smad molecules. (A, B)** MARK1 dysregulates time-resolved signaling dynamics of nuclear Smad3 and Smad2. Huh7 cells were used as a control. Cells were acutely stimulated with 5 ng/mL TGF-β. Nuclei were counterstained in blue with DAPI. Scale bar: 10 µm. MARK1, microtubule affinity regulating kinase 1; TGF-β, transforming growth factor-β.

**
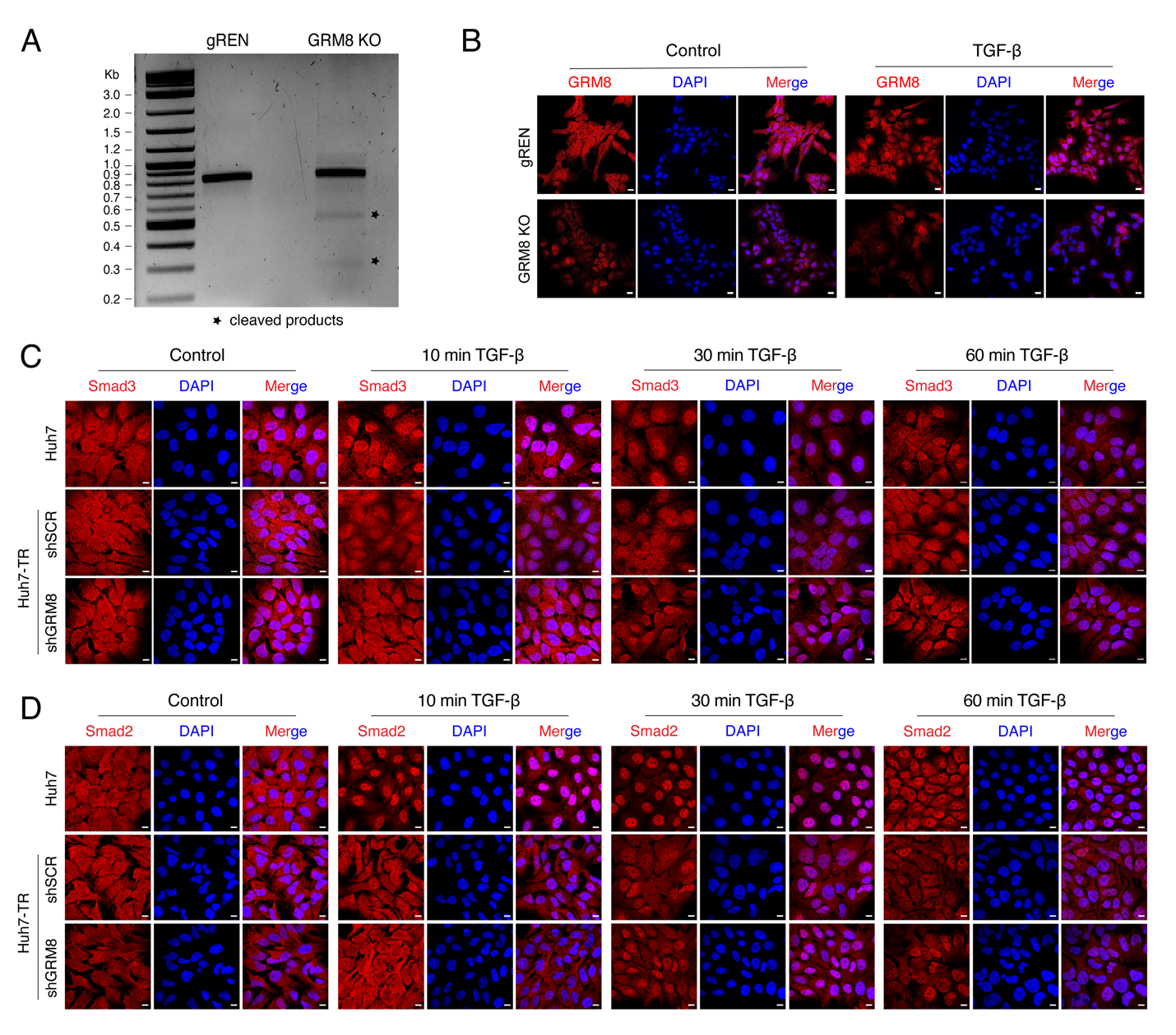
**

**Figure S13. GRM8 attenuates TGF-β/Smad Signaling. (A)** T7 Endonuclease I (T7E1) assay demonstrates genome editing in GRM8-targeted region. Molecular size marker is labeled. Star denotes cleaved products. **(B)** Representative images are shown for GRM8 immunofluorescence staining in gREN control and GRM8 KO clones. Scale bar: 20 µm. **(C, D)** GRM8 dysregulates time-resolved signaling dynamics of nuclear Smad3 and Smad2. Cells were acutely stimulated with 5 ng/mL TGF-β. Huh7 cells were used as a control. Nuclei were counterstained in blue with DAPI. Scale bar: 10 µm. GRM8, glutamate metabotropic receptor 8; TGF-β, transforming growth factor-β; KO, knockout.
