# Supplementary material for: Escape from TGF‐β‐induced senescence promotes aggressive hallmarks in epithelial hepatocellular carcinoma cells
Source: Mol Oncol. 2025 Mar 14;19(9):2594–618. doi: 10.1002/1878-0261.70021 (PMC12420363; doi:10.1002/1878-0261.70021)
Supplement: Supplementary file 2 — Table S1. Oligonucleotides for gRNA and shRNA cloning. Table S2. Primer sequences for qRT‐PCR. Table S3. Primary and secondary antibodies. Table S4. Top 50 genes in Cluster 2, Huh7‐TR vs Huh7. [file MOL2-19-2594-s001.docx]

**Supplementary Tables**

**Table S1. Oligonucleotides for gRNA and shRNA cloning**

| **Oligo** | **Sequence (5′-3′)** |
| --- | --- |
| Ren-gRNA-F | CACCGGTAGCGCGGTGTATTATACC |
| Ren-gRNA-R | AAACGGTATAATACACCGCGCTACC |
| MARK1-gRNA-F | CACCGCATTGCCGACGGTGAACGAG |
| MARK1-gRNA-R | AAACCTCGTTCACCGTCGGCAATGC |
| GRM8-gRNA-F | CACCGGTATGCCCATTCCATACGGG |
| GRM8-gRNA-R | AAACCCCGTATGGAATGGGCATACC |
| PLA2G4A-gRNA-F | CACCGACACCACTACCGTAAACTTG |
| PLA2G4A-gRNA-R | AAACCAAGTTTACGGTAGTGGTGTC |
| GRM8-shRNA-F | CCGGCTTGCCAATAATCGAAGAAATCTCGAGATTTCTTCGATTATTGGCAAGTTTTTG |
| GRM8-shRNA-R | AATTCAAAAACTTGCCAATAATCGAAGAAATCTCGAGATTTCTTCGATTATTGGCAAG |

**Table S2. Primer sequences for qRT-PCR**

| **Primer ID** | **Sequence (5′-3′)** |
| --- | --- |
| Smad2-F | CGAAATGCCACGGTAGAAAT |
| Smad2-R | GATTACAATTGGGGCTCTGC |
| Smad3-F | CAGAGTGCCTCAGTGACAGC |
| Smad3-R | AGCGAACTCCTGGTTGTTGA |
| Smad4-F | TTTGGGTCAGGTGCCTTAGT |
| Smad4-R | TGACACTGACGCAAATCAAAG |
| CDKN2B-F | ATGCGCGAGGAGAACAAG |
| CDKN2B-R | GAAACGGTTGACTCCGTTG |
| p21/ CDKN1A-F | CATGGGTTCTGACGGACATC |
| p21/ CDKN1A-R | TGCCGAAGTCAGTTCCTTGT |
| PAI-1-F | CACCCTCAGCATGTTCATTG |
| PAI-1-R | CCAGGTTCTCTAGGGGCTTC |
| p15-F | GCCCCAAGCCGCAGAAGGAC |
| p15-R | GCCCATCATCATGACCTGGATCGC |
| Nox4-F | TCCTCGGTGGAAACTTTTGT |
| Nox4-R | TGTCCCATATGAGTTGTTCTGG |
| CDH1-F | AGCGTGTGTGACTGTGAAGG |
| CDH1-R | CTCTTCTCCGCCTCCTTCTT |
| Vimentin-F | CGTCACCTTCGTGAATACCA |
| Vimentin-R | CCAGAGGGAGTGAATCCAGA |
| TGFβRI-F | TTGTGGCACGGTGAGAGTGT |
| TGFβRI-R | TGCTCCTGGGCTATTGAATCA |
| TGFβRII-F | GTTAACCGGCAGCAGAAGCT |
| TGFβRII-R | ATCAGCCAGTATTGTTTCCC |
| TGF-β1-F | TGCGGCAGCTGTACATTGA |
| TGF-β1-R | TGGTTGTACAGGGCCAGGA |
| GRM8-F | ACGGAGATGCTCCTGGACGTTA |
| GRM8-R | CTCTATGAGCCCACTGCATGTC |
| MARK1-F | TGCTCACCTGAAGGTCCAGAGA |
| MARK1-R | CTTTCCACACTGTTAGCCTGAGG |
| PLA2G4A-F | GGATTCTCTGGTGTGATGAAGGC |
| PLA2G4A-R | CCTTTCTCTGGAAAATCAGGGTG |
| GAPDH-F | GGCTGAGAACGGGAAGCTTGTCAT |
| GAPDH-R | CAGCCTTCTCCATGGTGGTGAAGA |
| β-Actin-F | TTCCTGGGCATGGAGTCCT |
| β-Actin-R | AGGAGGAGCAATGATCTTGATC |

**Table S3. Primary and secondary antibodies**

| **Antibodies** | **Commpany and catalog number** | **Western blot working dilution** | **Immunostaining working dilution** |
| --- | --- | --- | --- |
| p-pRb | Cell Signaling, 9308 | 1:1000 (O/N) | - |
| p15 | Santa Cruz, sc612 | 1:400 (RT)  1:1000 (O/N) | - |
| p21 | Calbiochem, OP64 | 1:100 (RT)  1:250 (O/N) | - |
| PAI-1 | Santa Cruz, sc5297 | 1:500 (O/N) | - |
| Nox4 | Santa Cruz, sc30141 | 1:2500 (O/N)  1:750 (RT) | - |
| α-tubulin | Calbiochem, CP06 | 1:10000 (RT)  1:25000 (O/N) | - |
| e-cadherin | Santa Cruz, sc7870 | 1:2000 (O/N) | - |
| Vimentin | Dako, M7020 | 1:5000 (O/N) | 1:500 |
| ZO-1 | Transduction, 610966 | 1:2500 (O/N) | 1:500 |
| β-catenin | Santa Cruz, sc7963 | - | 1:350 |
| p-Smad3 | Cell Signaling, 9520 | 1:1000 (O/N) | 1:150 |
| p-Smad3/Smad1 | Cell Signaling, 9514 | 1:1000 (O/N) | - |
| Smad3 | Cell Signaling, 9523 | 1:1000 (O/N) | 1:200 |
| Smad2/3 | BD Biosciences 610843 | - | 1:250 |
| Smad2/3 | Cell Signaling, 8685 | 1:1000 (O/N) | - |
| Smad4 | Santa Cruz, sc7966 or  Cell Signaling, 38454 | 1:1000 (O/N) | 1:250 |
| PARP | Santa Cruz, sc8007 | 1:1000 (O/N) | - |
| β- actin | Cell Signaling, 3700 or  Santa Cruz, sc1616 | 1:5000 (O/N) or  1:2000 (O/N) | - |
| GSK3β | Transduction, G22320 | 1:2500 (O/N) | - |
| p-Smad2 | Cell Signaling, 3108 | 1:1000 (O/N) | - |
| Smad2 | Cell Signaling, 5339 | 1:1000 (O/N) | 1:200 |
| MARK1 | Proteintech, 21552-1-AP | 1:1000 (O/N) | - |
| cPLA2 | Santa Cruz, sc454 | 1:200 (O/N) + RT | - |
| Anti-Flag | Millipore, F7425 | 1:2000 (O/N) | - |
| GRM8 | Proteintech, 18224-1-AP | - | 1:200 |
| CK19 | Santa Cruz, sc6278 | - | 1:250 |
| EpCAM | Santa Cruz, sc25308 | - | 1:250 |
| Lamin A/C | Abcam, ab190380 | - | 1:300 |
| anti-mouse IgG (H+L) (DyLightTM 680 Conjugate) | Cell Signaling, 5470 | 1:10000 (RT) | - |
| anti-rabbit IgG (H+L) (DyLightTM 800 4X PEG Conjugate) | Cell Signaling, 5151 | 1:20000 (RT) | - |
| Dnk pAb to Ms IgG Alexa Flour 488 | Abcam, ab150105 | 1:1000 (RT) |  |
| Anti-mouse IgG (H+L), F(ab')2 Fragment (Alexa Fluor® 594 Conjugate) | Cell Signaling, 8890 | 1:1000 (RT) |  |

**Table S4. Top 50 genes in Cluster 2, Huh7-TR vs Huh7.**

| **Gene name** | **log_2_FC** | **p-value** |
| --- | --- | --- |
| MARK1 | 7.74806822 | 2.1412E-39 |
| ST6GALNAC3 | 6.77881501 | 4.1415E-24 |
| PLA2G4A | 5.88983434 | 8.2519E-64 |
| SSTR1 | 5.60893744 | 4.3161E-50 |
| ADAMTS3 | 5.4785106 | 3.1964E-48 |
| GRM8 | 5.26065004 | 1.0762E-71 |
| CLIC6 | 5.2312693 | 6.906E-28 |
| PGC | 4.85507909 | 6.4922E-49 |
| VEPH1 | 4.43318478 | 1.1416E-19 |
| SPINK4 | 4.29021981 | 5.2791E-06 |
| MCOLN3 | 4.21733152 | 2.1633E-35 |
| NAALADL2 | 4.03048416 | 3.1858E-09 |
| ERBB4 | 3.98030831 | 2.1385E-09 |
| COL11A1 | 3.92541574 | 5.6775E-12 |
| CD38 | 3.84214334 | 6.0005E-21 |
| CADM2 | 3.8390611 | 1.5689E-22 |
| ZNF462 | 3.64833562 | 1.0863E-39 |
| PZP | 3.62827482 | 9.2215E-13 |
| CCNI2 | 3.5262438 | 9.1516E-09 |
| IGSF10 | 3.4211852 | 2.4423E-26 |
| CPNE4 | 3.36815129 | 1.0896E-10 |
| PTX3 | 3.34386916 | 1.4479E-12 |
| CPED1 | 3.31814448 | 2.1585E-14 |
| LAMA3 | 3.24396058 | 1.0659E-21 |
| SEMA3E | 3.1812164 | 2.4566E-21 |
| FUT3 | 3.11076187 | 2.7401E-10 |
| EFNA5 | 3.05468224 | 1.0946E-28 |
| ADAMTS12 | 3.00316679 | 9.8114E-88 |
| PYGO1 | 2.97750119 | 1.2586E-38 |
| BRINP3 | 2.97074827 | 1.528E-16 |
| TRPC6 | 2.85409439 | 9.6703E-11 |
| TRIM9 | 2.85061612 | 2.8788E-15 |
| CTSE | 2.8341352 | 1.8416E-66 |
| RALYL | 2.80363571 | 1.2292E-10 |
| SLC25A12 | 2.80116496 | 1.1857E-09 |
| E2F7 | 2.73037038 | 3.4683E-52 |
| ARHGAP24 | 2.71389793 | 2.4676E-32 |
| CPA2 | 2.63207139 | 1.9201E-16 |
| ENPP5 | 2.62591834 | 2.3274E-07 |
| HMCN1 | 2.62568433 | 2.028E-24 |
| ENPP4 | 2.55984386 | 2.9006E-46 |
| CHSY3 | 2.5427783 | 4.2289E-31 |
| MN1 | 2.54193216 | 3.0178E-10 |
| PCDHB5 | 2.52771845 | 2.2334E-44 |
| OSBPL1A | 2.50995501 | 2.9079E-15 |
| KCNJ8 | 2.46009546 | 3.1181E-35 |
| PLA1A | 2.44463316 | 1.8023E-10 |
| CYP17A1 | 2.42270866 | 1.7071E-08 |
| COL4A5 | 2.37732182 | 6.58E-62 |
| BIRC3 | 2.36126222 | 1.7456E-10 |
